# Overexpression of cyclin F/*CCNF* as an independent prognostic factor for poor survival in clear cell renal cell carcinoma

**DOI:** 10.1038/s41598-024-59437-1

**Published:** 2024-04-23

**Authors:** Maciej Kwiatkowski, Adrian Krajewski, Justyna Durślewicz, Karolina Buchholz, Dariusz Grzanka, Maciej Gagat, Jan Zabrzyński, Anna Klimaszewska-Wiśniewska

**Affiliations:** 1 Department of Clinical Pathomorphology, Faculty of Medicine, Collegium Medicum in Bydgoszcz, Nicolaus Copernicus University in Toruń, Bydgoszcz, Poland; 2 Department of Urology and Urological Oncology, Multidisciplinary Hospital of Ludwik Blażek, Inowrocław, Poland; 3 Department of Histology and Embryology, Faculty of Medicine, Collegium Medicum in Bydgoszcz, Nicolaus Copernicus University in Toruń, Bydgoszcz, Poland; 4 Department of Orthopaedics and Traumatology, Faculty of Medicine, Collegium Medicum in Bydgoszcz, Nicolaus Copernicus University in Toruń, Bydgoszcz, Poland; 5 Faculty of Medicine, Collegium Medicum, Mazovian Academy, Płock, Poland

**Keywords:** Biomarkers, Diseases, Oncology

## Abstract

Cyclin F (encoded by *CCNF* gene) has been reported to be implicated in the pathobiology of several human cancers. However, its potential clinical significance in clear cell renal cell carcinoma (ccRCC) remains unknown. The present study aimed to evaluate the potential significance of cyclin F, assessed by immunohistochemical (IHC) staining and molecular (bioinformatics) techniques, as a prognostic marker in ccRCC in relation to clinicopathological features and outcomes. IHC staining was performed using two independent ccRCC tissue array cohorts, herein called tissue macroarray (TMA)_1 and tissue microarray (TMA)_2, composed of 108 ccRCCs and 37 histologically normal tissues adjacent to the tumor (NAT) and 192 ccRCCs and 16 normal kidney samples, respectively. The mRNA expression data were obtained from The Cancer Genome Atlas (TCGA) and the Gene Expression Omnibus (GEO) public datasets, followed by bioinformatics analysis of biological mechanisms underlying prognosis. The relationship between immune cell infiltration level and *CCNF* expression in ccRCC was investigated using the Tumor Immune Estimation Resource 2.0 (TIMER2) and Gene Expression Profiling Interactive Analysis 2 (GEPIA2). Cyclin F expression was significantly elevated in ccRCC lesions compared to both NAT and normal renal tissues. Likewise, *CCNF* mRNA was markedly increased in ccRCCs relative to non-cancerous tissues. In all analyzed cohorts, tumors with features of more aggressive behavior were more likely to display cyclin F/*CCNF*-high expression than low. Furthermore, patients with high cyclin F/*CCNF* expression had shorter overall survival (OS) times than those with low expression. In addition, multivariable analysis revealed that cyclin F/*CCNF*-high expression was an independent prognostic factor for poor OS in ccRCC. Enrichment analysis for mechanistically relevant processes showed that *CCNF* and its highly correlated genes initiate the signaling pathways that eventually result in uncontrolled cell proliferation. *CCNF* expression was also correlated with immune cell infiltration and caused poor outcomes depending on the abundance of tumor-infiltrating immune cells in ccRCC. Our findings suggest that cyclin F/*CCNF* expression is likely to have an essential role in ccRCC pathobiology through regulating multiple oncogenic signaling pathways and affecting the tumor immune microenvironment and may serve as prognostic biomarker and promising therapeutic target in ccRCC.

## Introduction

Renal cell carcinoma (RCC) accounts for approximately 2% of cancer-related diagnoses and deaths globally. Its incidence rate has increased in the last decades, attributed to incidental diagnoses associated with the extensive use of imaging techniques^1,2^. Clear-cell RCC (ccRCC) represents the predominant and simultaneously the most lethal histological subtype of RCC tumors^3^. When diagnosed early, ccRCC can be successfully managed with surgery. Nevertheless, due to its typically asymptomatic nature, nearly 28% of cases are initially diagnosed with regional or distant metastases^4^.

Moreover, up to 30% of the patients with initially localized disease experience recurrence after cancer resection^5,6^, and the high heterogeneity of ccRCC as well as its resistance to radiotherapy and chemotherapy, renders available systemic therapies largely ineffective^7^. Consequently, 5-year overall survival in the IV stage of ccRCC is 19.7%^8^. In the face of difficulties in the early detection of ccRCC and the inadequate effectiveness of non-surgical methods, identifying new reliable diagnostic and prognostic biomarkers seems crucial for improving patient outcomes.

Cyclin F, also known as F-box only protein 1 (Fbxo1), unlike the other cyclins, does not act as an activator of cyclin-dependent kinases (CDKs). Instead, it is a member of the F-box proteins being substrate recognition subunits of SCF (Skp1-Cul1-F-box protein) ubiquitin ligase complexes. Consequently, cyclin F is implicated in various target proteins’ ubiquitination and subsequent degradation. The protein level of cyclin F increases in the S phase and peaks in the G2 phase of the cell cycle, which is strictly regulated to ensure the scheduled degradation of substrates^9^. The altered expression of cyclin F has been shown to be associated with poor prognosis of patients with various types of cancers^10–14^ as well as the growth, metastasis, and drug resistance in some of them. Considering these findings, cyclin F emerges as a potential prognostic marker and therapeutic target. However, its clinical significance in ccRCC remains unexplored.

In this paper, we explore the clinical association of cyclin F/CCNF with ccRCC pathology and outcome using two independent tissue array ccRCC cohorts and TCGA ccRCC cohort, followed by further validation of the findings with additional bioinformatics analyses. In addition, the mechanisms underlying how *CCNF* affects ccRCC were examined by analyzing the correlations between *CCNF* and other genes followed by functional enrichment analysis.

## Materials and methods

### Ethics statement

The usage of archived diagnostic leftover tissues for research purposes, as well as patient data analysis, was approved by the Bioethics Committee of the Nicolaus Copernicus University in Toruń functioning at Collegium Medicum in Bydgoszcz (no. 253/2018). The need for informed consent was waived by the same Bioethics Committee as mentioned above (Bioethics Committee of the Nicolaus Copernicus University in Toruń functioning at Collegium Medicum in Bydgoszcz) in view of the retrospective nature of the study and all procedures performed were part of routine care, and the study utilized only leftover tissues, with the analysis using anonymous clinical data*.* Data collection and management were performed in compliance with the principles of the Declaration of Helsinki.

### Tissue arrays and immunochemistry staining

The study was conducted retrospectively in two independent tissue arrays (TAs) cohorts of ccRCC patients. The first one consisted of 108 ccRCC cases and 37 adjacent non-tumor tissues obtained from the patients who underwent surgery in the Department of Urology and Andrology, Antoni Jurasz University Hospital No. 1 in Bydgoszcz (Poland) and were subsequently diagnosed with ccRCC in the Department of Clinical Pathomorphology, Collegium Medicum in Bydgoszcz of Nicolaus Copernicus University in Torun; whereas the other one was commercially purchased (tissue microarray (TMA) no. KD20810a, TissueArray.Com LLC, Derwood, MD, USA) and contained 192 ccRCC tumors and 16 normal kidney samples (1 mm diameter spot per patient). Tissue macroarrays (TMAs) for the former cohort were constructed according to the previously described method^10^. Each paraffin recipient block containing five different large (about 5–7 µm) tissue fragments was sectioned at 4-μm thickness and placed on high-adhesive glass slides (SuperFrost Plus; Menzel-Glaser, Braunschweig, Germany). The institutional tissue macroarray cohort was thereafter referred to as the TMA_1 cohort, while the commercial microarray cohort was called TMA_2. Tissue sections of both cohorts were stained by the same previously described procedure^10^. They were incubated with a primary rabbit polyclonal anti-cyclin F antibody (1:100, 40 min; cat. no: HPA071600, Merck Millipore, Burlington, MA, USA) on BenchMark ULTRA system (Roche Diagnostics/Ventana Medical Systems, Tucson, AZ, USA). Antigen–antibody reactions were visualized using an ultraView Universal DAB Detection Kit (Roche Diagnostics/Ventana Medical Systems, Tucson, AZ, USA). The complete clinicopathological characteristics of experimental groups are presented in Supplementary Table S1 online. Overall survival (OS) was defined as the time interval between the date of surgery and date of death. OS data were censored if patients were alive at the last follow-up date (21 January 2021). The median follow-up time (calculated by the reverse Kaplan–Meier method) and median OS time for patients in the TMA_1 cohort were 181 and 42 months, respectively.

### Evaluation of immunostaining

TMA sections were examined for immunoreactivity and graded semi-quantitatively using the H-score system by three investigators in a blinded fashion under a multi-headed microscope (Olympus, Tokyo, Japan) at 20 × original objective magnification. The H-score was calculated by adding the products of the percentage of cells stained at a given staining intensity (0–100) and the staining intensity score (1, weak, 2, moderate, and 3, strong), resulting in a range from 0 to 300. The H-score was calculated as follows: ∑ (pi × i) = (1 × percentage of weak intensity) + (2 × percentage of moderate intensity) + (3 × percentage of strong intensity). To determine "high" or "low" expression levels of cyclin F in the TMA_1 cohort, the final H-scores were dichotomized based on the optimal cut-off point, as well as the median (H-score ≤ 21.5, low expression; H-score > 21.5, high expression; data provided in Supplementary files). The optimal cut-off value for cyclin F expression was determined using the *cutp* algorithm in the Evaluate Cupoints software^15^ and classified as follows: H-score < 11, low expression; H-score ≥ 11, high expression. Patients of the TMA_2 cohort were divided into a low cyclin F expression group (H-score ≤ 5) and high cyclin F expression group (H-score > 5) based on the median value of the H-score.

### Extraction of RNA-sequencing TCGA data and clinical information

The Cancer Genome Atlas (TCGA) and Genotype-Tissue Expression (GTEx) databases provided data on *CCNF* expression for 475 ccRCC primary tumors and 28 normal tissue samples, respectively. The baseline characteristics of these patients are presented in Supplementary Table S1. The above data, normalized using the DESeq2 method, were downloaded through the University of California Santa Cruz (UCSC) Xena Browser (https://xenabrowser.net/, accessed on 14 September 2022)^16^, whereas corresponding clinical information was retrieved from cBioPortal (https://www.cbioportal.org, accessed on 14 September 2022). According to the  optimal cutpoint determined for *CCNF* expression levels using the *cutp* function of the Evaluate Cutpoints software^15^, ccRCC patients were divided into high (≥ 7.884) and low (< 7.884) expression groups. The median OS time for patients in the TCGA cohort was 116 months.

### TNMplot analysis

The TNMplot database (http://tnmplot.com; accessed on 28 November 2022) was applied to compare the *CCNF* gene expression in paired tumor and adjacent normal tissues, as well as between normal, tumor, and metastatic tissues. This analysis utilized the gene chip data obtained from the National Center for Biotechnology Information Gene Expression Omnibus Database (NCBI-GEO)^17^. Besides, *CCNF* expression levels were also compared in TCGA tumor samples paired with adjacent TCGA normal samples based on RNA-seq data. The results were visualized by violin plots. *CCNF* expression in normal, tumor, and metastatic tissues was compared using Kruskal–Wallis test with Dunn's post hoc tests, whereas the difference between two groups (tumor vs. control tissue) was analyzed with a Mann–Whitney U test or a paired Wilcoxon statistical test.

### ShinyGEO

The ShinyGEO (http://gdancik.github.io/shinyGEO/, accessed on 30 November 2022) was used for further differential gene expression analysis, where GSE15641, GSE16449, GSE66272, GSE1963, and GSE105261 were employed^18^. Boxplots were used to depict the distribution of gene expression levels. The Mann–Whitney or Wilcoxon test or Kruskal–Wallis test with Dunn's post hoc test was performed to assess whether the expression difference was statistically significant (p < 0.05).

### UALCAN web portal

The "TCGA" module of the UALCAN online tool (http://ualcan.path.uab.edu/, accessed on 30 November 2022) was used to assess the relative expression of *CCNF* in different grades of kidney renal clear cell carcinoma (KIRC)^19^. The results were presented in box-whisker plots, and differences in transcriptional expression were compared by Student's t-test considering unequal variance. *P* < 0.05 was considered statistically significant.

### GENT2, GEPIA2, Kaplan plotter online databases

GENT2 and GEPIA2 online tools were used to validate the prognostic value of *CCNF* expression in RCC cohorts derived from the NCBI-GEO public database and TCGA, respectively. Meta-survival analysis of OS with Cox proportional hazard modes was done using the data from the Gene Expression across Normal and Tumor tissue (GENT2) (http://gent2.appex.kr/gent2/; accessed on 29 November 2022) and depicted as a forest plot^20^. The Gene Expression Profiling Interactive Analysis (GEPIA2, http://gepia2.cancer-pku.cn/, accessed on 13 December 2022) was used to obtain the OS plots of the candidate gene across KIRC-TCGA tumors^21^. Median and quartile values [cutoff-high (50%, 75%, respectively) and cutoff-low (50%, 25%, respectively)] were set as the expression thresholds for splitting the high-expression and low-expression cohorts. Survival curves with the calculated HRs with 95% CIs and the log-rank p-values were plotted. GEPIA2-based analysis was also used to determine the correlation between *CCNF* expression and immune infiltration associated markers (accessed on 8 December 2023). The correlation was analyzed by Spearman's correlation coefficient. In addition, the online Kaplan–Meier Plotter tool (http://kmplot.com/analysis, accessed on 13 December 2022), which combines survival data from GEO, TCGA, and other databases, was applied further to confirm the effect of *CCNF* on ccRCC prognosis^22^. Kaplan–Meier curves were plotted using the "mRNA RNA-seq" module and "*CCNF*" as input queries; patients were divided based on the best cut-off and median. Analyses were or were not filtered by tumor stage. A statistically significant difference was considered when a p-value < 0.05.

### TIMER2 database analysis

In the present study, we conducted the Tumor IMmune Estimation Resource 2.0 (TIMER2; accessed on 8 December 2023)^23^ to confirm the expression and prognostic value of *CCNF* in TCGA-KIRC. Moreover, we applied TIMER2 to determine the correlation between *CCNF* expression and the abundance of tumor-infiltrating immune; however, we focused only on those immune cells that were significantly associated with the overall survival via the ‘Outcome_Module’ of TIMER2. The ‘Outcome_Module’ was also used to explore the difference in overall survival among patients stratified by both the estimated infiltration level of immune cells and *CCNF* expression level in KIRC. Given that the tumor purity of the sample impacts the immuno-infiltration analysis, the correlation analysis was adjusted accordingly. All analyses were performed with default parameters in TIMER2 and p-value < 0.05 was considered statistically significant.

### PPI construction and functional enrichment analysis

The list of genes positively and negatively correlated with *CCNF* in ccRCC was retrieved from the UALCAN database (http://ualcan.path.uab.edu/, accessed on 23 March 2023)^19^. The top positively correlated genes (Pearson CC values ≥ 0.7) and all negatively correlated genes (Pearson CC values ≥ 0.3) were selected to construct the zero-order protein–protein interaction network (PPI) using The Search Tool for the Retrieval of Interacting Genes Database (STRING)^24^ and Cytoscape software (version 3.9.1). The top 10 genes in the network were screened as hub genes using three topological analysis methods of the CytoHubba plugin (version 0.1) in the Cytoscape software^25^. The Molecular Complex Detection (MCODE) clustering algorithm (version 2.0.2) was then used to identify clustered modules within the PPI network according to the following criteria: degree cut-off = 2, max. depth = 100, k-core = 2, haircut = yes, and node score cutoff = 0.2^26^. The most significant subnetwork was tested for overrepresentation of the Gene Ontology (GO) terms using the DAVID Functional Annotation Clustering Tool (The Database for Annotation, Visualization and Integrated Discovery version 6.8, DAVID; https://david.ncifcrf.gov, accessed on 23 March 2023)^27^, whereas the ClueGO plugin (version 2.5.9) was used to identify enriched KEGG and Reactome pathways (with a threshold of p ≤ 0.0001 based on a two-sided hypergeometric test (kappa score 0.4) and the Bonferroni correction)^28^.

### Statistical analysis

All statistical analyses were conducted using GraphPad Prism (version 8.0, GraphPad Software, San Diego, CA, USA), SPSS (version 28.0, IBM Corporation, Armonk, NY, USA), or Rstudio (version 1.3.1093) software packages. Data distribution was determined with the Shapiro–Wilk test, and the appropriate parametric or nonparametric statistical tests were applied. The Mann–Whitney or Wilcoxon signed-rank test was performed to compare differences in cyclin F expression between experimental and control groups. The chi-square or Fisher's exact test was employed to evaluate differences between cyclin F expression status and clinicopathological parameters. The Kaplan–Meier curves and log-rank test were utilized to compare survival outcomes in different groups of ccRCC patients. The prognostic ROC curves were constructed from Kaplan–Meier survival estimates, following the method described by Combescure et al.^29^. Univariable and multivariable analyses by Cox proportional hazards method were performed to establish the risk factors for the overall survival. Crude and adjusted hazard ratios (HRs) with a relative 95% confidence interval were shown. A backward elimination procedure built all multivariable models, with a significance level of p < 0.05 to enter the model and p < 0.10 to stay. The proportional hazard assumptions were verified graphically using plots of log(−log) survival and Schoenfeld residuals. Covariates violating the PH assumption were introduced as time-dependent covariates in the Cox regression models. All reported p-values were two-sided with a significance level of 0.05.

## Results

### Expression levels of cyclin F/*CCNF* in ccRCC and control tissues

We first determined the IHC expression of cyclin F in two independent TMA cohorts: in-house cohort (TMA_1), consisting of 108 ccRCC tissues and 37 adjacent non-tumor tissues, and the other, commercially purchased one (TMA_2), encompassing 192 ccRCC cases and 16 samples of normal renal tissue. After excluding non-representative and missing cores, IHC results were available for 108 (100%) and 172 (89.58%) cases of TMA_1 and TMA_2 cohorts, respectively.

**Figure 1:**
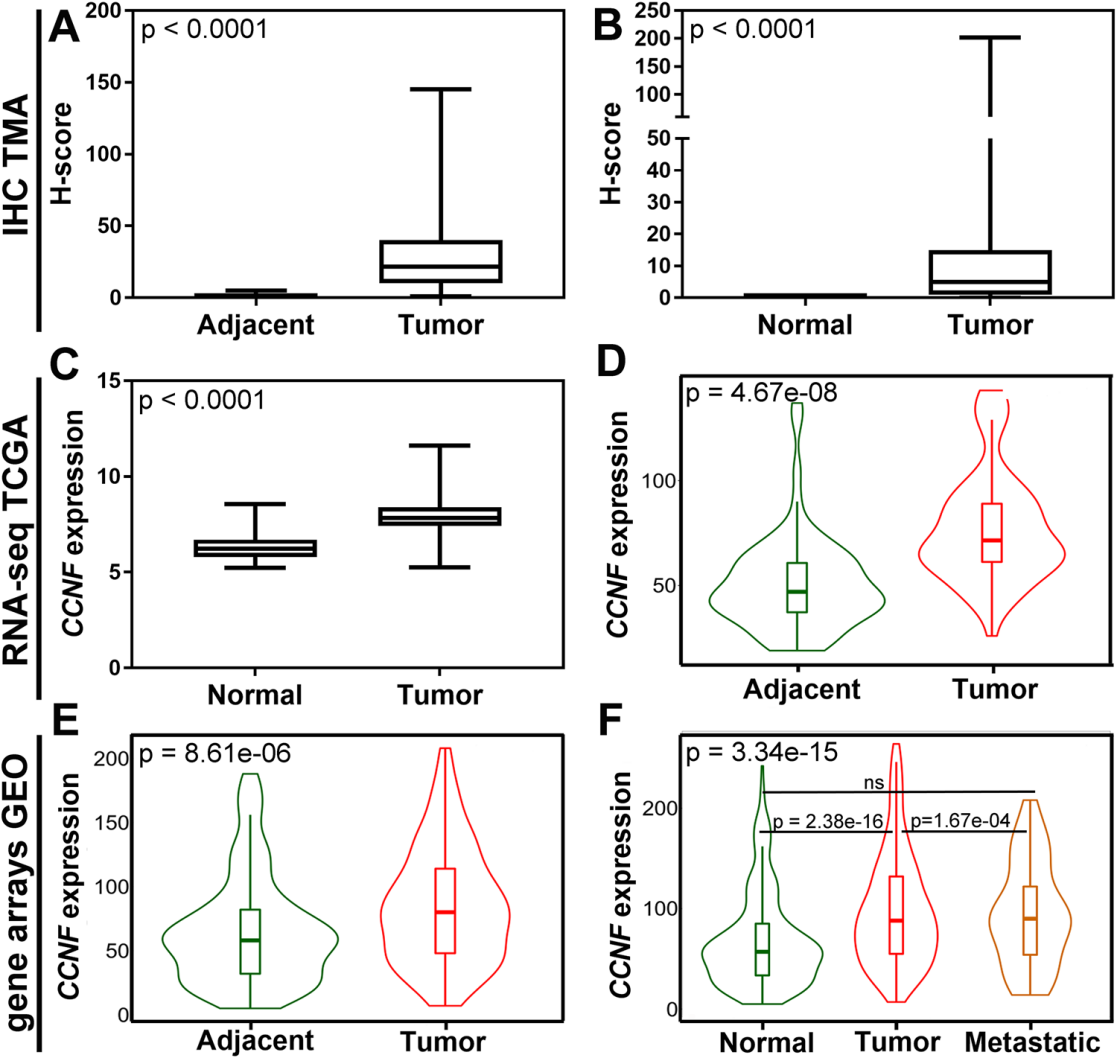
Expression of cyclin F/*CCNF* in clear cell renal cell carcinoma compared to non-cancerous control tissue. Boxplot graphs of (**a**) cyclin F expression in tumor tissue (n = 108) and histologically normal tissue adjacent to the tumor (n = 37) of the institutional tissue macroarray (TMA) cohort based on immunohistochemistry (IHC); (**b**) cyclin F expression in tumor tissue (n = 172) and normal renal tissue (n = 16) of the commercially purchased microarray (TMA) cohort based on IHC; (**c**) *CCNF* expression levels in tumor tissue (n = 475) and normal renal tissue (n = 27) based on RNA-Seq data from The Cancer Genome Atlas (TCGA) and Genotype-Tissue Expression (GTEx) databases, respectively, downloaded from UCSC Xena Browser. Violin plots from the TNMplot web tool showing (**d**) *CCNF* expression levels in paired tumor tissue (n = 72) and adjacent normal tissue (n = 72) based on RNA-Seq data from the TCGA; (**e**) *CCNF* expression levels in paired tumor tissue (n = 210) and adjacent normal tissue (n = 210) based on the gene chip data derived from the Gene Expression Omnibus (GEO) database. (**f**) *CCNF* expression levels in primary tumor tissue (n = 556), non-paired normal tissue (n = 277), and metastasis (n = 58) based on the gene chip data derived from the GEO database.

Faint cytoplasmic staining of cyclin F was occasionally observed in a renal tubular epithelium of adjacent non-tumor tissues but not in a renal tubular epithelium of normal tissues (Supplementary Fig. S1). In control tissues, mild to moderate cyclin F labeling was noticed in endothelial cells of glomerular capillaries and, to a lesser frequency, peritubular capillaries, as opposed to the much more common and varied in intensity (often strong) staining of endothelial cells of tumor-associated vasculature (Supplementary Fig. S1). In the tumor tissues of both cohorts, cyclin F staining was predominantly cytoplasmic, with mixed membranous-cytoplasmic staining or solely membranous staining observed in the same cases (Supplementary Fig. S1). In addition, we identified noticeable inter- and intratumoral differences in IHC staining intensity, distribution, and patterns, which could be partially due to the glycogen and lipid-rich cytoplasmic deposits being washed away during tissue processing. Statistical analysis showed a significant increase of cyclin F expression in ccRCC compared to tumor-adjacent normal tissue (Fig. 1a) or normal renal tissue (Fig. 1b). According to the established cut-off points, high expression levels of cyclin F were found in 74 (68.52%) and 78 (45.35%) cases of ccRCC in the TMA_1 and TMA_2 cohorts, respectively.

Consistent with the protein data, the RNA-Seq data from the TCGA showed that the *CCNF* expression was significantly increased in ccRCC tissues relative to normal renal tissues (Fig. 1c). Similar results were obtained from the TNMplot online tool based on the TCGA-RNA-seq data when comparing paired ccRCC and histologically normal tissue adjacent to the tumor (Fig. 1d). Likewise, *CCNF* expression was higher in tumor tissues than in matched adjacent normal tissues (Fig. 1e) or non-paired normal tissue (Fig. 1f) but not in metastasis (Fig. 1f) from the gene chip data of the TNMplot. Notably, *CCNF* expression in metastases was significantly lower by a factor of 0.85 relative to primary tumors (Fig. 1f). However, a differential expression analysis of individual GEO datasets through the ShinyGEO web-based application found that *CCNF* expression was increased when compared to control and tumor tissues and between primary tumor and metastatic tissues; nevertheless, in the latter case, the statistical significance was not reached (p = 0.134; Supplementary Fig. S2).

### Association with clinicopathological features

In the TMA_1 cohort (Table 1), ccRCCs with increasing tumor grade more frequently had cyclin F-high than cyclin F-low expression (p = 0.018). Furthermore, high-level cyclin F expression was marginally significantly linked to more advanced pT stages (p = 0.055). No other associations were found between cyclin F and investigated clinicopathological parameters.

**Table 1:** Associations of cyclin F with clinicopathological features in the TMA cohorts.

Variable	n (%)	Cyclin F/TMA_1_		*p*	Variable	n (%)	Cyclin F/TMA_2		*p*
		Low n = 34	High n = 74				Low n = 94	High n = 78	
Age					Age				
≤ 60	45 (41.67)	13 (28.89)	32 (71.11)	0.678	≤ 60	105 (61.05)	56 (53.33)	49 (46.67)	0.754
> 60	63 (58.33)	21 (33.33)	42 (66.67)		> 60	67 (38.95)	38 (56.72)	29 (43.28)	
Sex					Sex				
Male	75 (69.44)	26 (34.67)	49 (65.33)	0.370	Male	115 (66.86)	62 (53.91)	53 (46.09)	0.871
Female	33 (30.56)	8 (24.24)	25 (75.76)		Female	57 (33.14)	32 (56.14)	25 (43.86)	
Grade					Grade				
G1	24 (22.22)	13 (54.17)	11 (45.83)	**0.018**	Gx	8 (4.65)	–	–	**0.017**
G2	71 (65.74)	19 (26.76)	52 (73.24)		G1	138 (80.23)	80 (57.97)	58 (42.03)	
G3–G4	13 (12.04)	2 (15.38)	11 (84.62)		G2–G3	26 (15.12)	8 (30.77)	18 (69.23)	
pT status					pT status				
T1	34 (31.48)	16 (47.06)	18 (52.94)	0.055	T1	117 (68.02)	70 (59.83)	47 (40.17)	0.051
T2	29 (26.85)	8 (27.59)	21 (72.41)		T2–T4	55 (31.98)	24 (43.64)	31 (56.36)	
T3–T4	45 (41.67)	10 (22.22)	35 (77.78)		–	–	–	–	
pN status					TNM stage				**0.034**
N0	101 (93.52)	33 (32.67)	68 (67.33)	0.429	I	118 (68.60)	71 (60.17)	47 (39.83)	
N+	7 (6.48)	1 (14.29)	6 (85.71)		II–IV	54 (31.40)	23 (42.59)	31 (57.41)	

Significant values are in bold.

In the TMA_2 cohort (Table 1), ccRCC patients with histologic grade 2 and 3 tumors more frequently had cyclin F-high expression (69.23%) than low expression (30.77%; p = 0.017). Furthermore, pT2-pT4 tumors tended to be cyclin F high expressors (56.36%) than cyclin F low expressors (43.64%), while in pT1 tumors, it was the opposite (40.17% vs. 59.83%; p = 0.051). A similar association was found regarding the TNM stage (p = 0.034). No other relationships were observed between cyclin F and clinicopathological features.

In the TCGA cohort (Table 2), ccRCC patients affected by advanced T and TNM stage (both p < 0.0001), or those with a proven positive lymph node (p = 0.014), or M1 disease (p < 0.0001) more frequently had tumors with *CCNF*-high expression than *CCNF*-low expression. Moreover, there was a trend toward the association of *CCNF* status with tumor grade (p = 0.107). Notably, when analyzed as a continuous variable using the UALCAN web tool, *CCNF* expression increased with increasing tumor grade (Supplementary Fig. S3). Statistical significance was reached between the subgroups, except for grade 1 vs. grade 2 tumors (Supplementary Fig. S3). No other significant associations existed between the tested biomarker and remaining clinicopathological features, including age and sex.

**Table 2:** Associations of *CCNF* with clinicopathological features in the TCGA cohort*.*

Variable	n (%)	*CCNF* expression		*p*
		Low n = 257	High n = 218	
Age (years)				
≤ 60	239 (50.32)	126 (52.72)	113 (47.28)	0.581
> 60	236 (49.68)	131 (55.51)	105 (44.49)	
Sex				
Male	312 (65.68)	160 (51.28)	152 (48.72)	0.099
Female	163 (34.32)	97 (59.51)	66 (40.49)	
Grading				
G1	11 (2.32)	8 (72.73)	3 (27.27)	0.107
G2	203 (42.74)	118 (58.13)	85 (41.87)	
G3–G4	261 (54.95)	131 (50.19)	130 (49.81)	
pT status				
T1	237 (49.89)	154 (64.98)	83 (35.02)	**< 0.0001**
T2	61 (12.84)	38 (62.30)	23 (37.70)	
T3–T4	177 (37.26)	65 (36.72)	112 (63.28)	
pN status				
Nx	235 (49.47)	–	–	
N0	225 (47.37)	124 (55.11)	101 (44.89)	**0.014**
N1	15 (3.15)	3 (20.00)	12 (80.00)	
pM status				
Mx	15 (3.16)	–	–	**< 0.0001**
M0	391 (82.32)	226 (57.80)	165 (42.20)	
M1	69 (14.52)	19 (27.54)	50 (72.46)	
TNM stage				
I	234 (49.26)	153 (65.38)	81 (34.62)	**< 0.0001**
II	50 (10.53)	33 (66.00)	17 (34.00)	
III	119 (25.05)	51 (42.86)	68 (57.14)	
IV	72 (15.15)	20 (27.78)	52 (72.22)	

Significant values are in bold.

### Association with the clinical outcome

**Figure 2:**
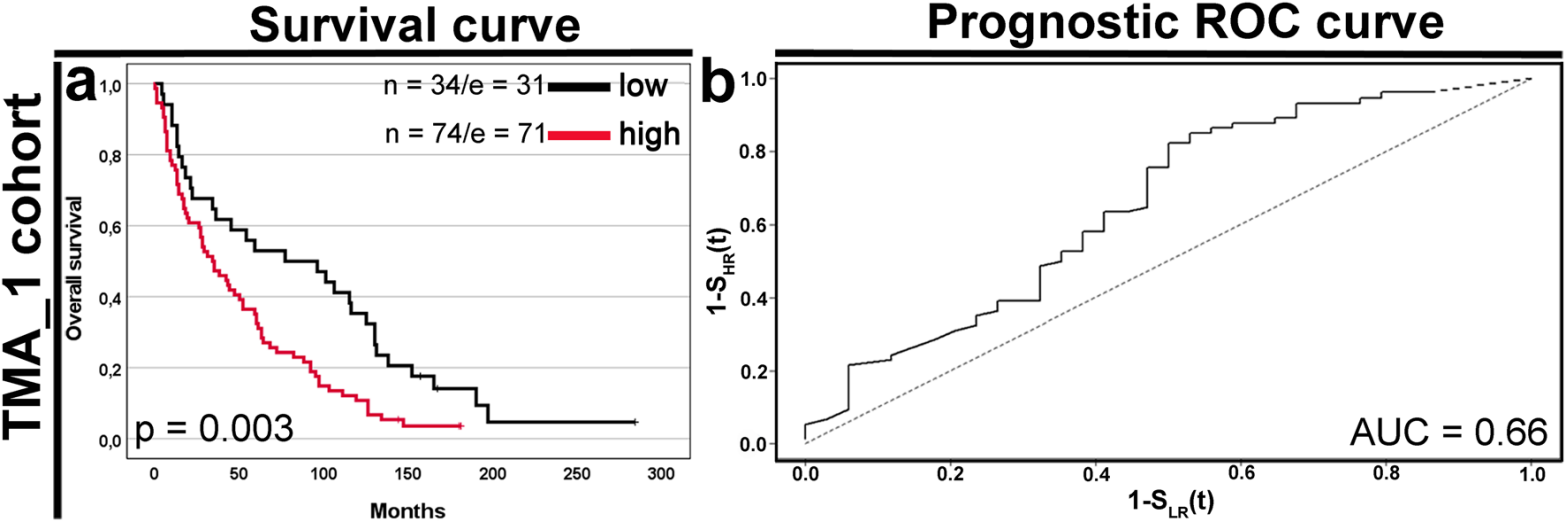
Effect of cyclin F expression on survivals in clear cell renal cell carcinoma cohort. Cases were divided into low and high-expression groups according to the optimal cut-off point (C_p_ = 11) determined by the Evaluate Cutpoints software. (**a**) Kaplan–Meier curve comparing high and low expression of cyclin F in the entire TMA_1 cohort (n = 108) and the corresponding (**b**) prognostic ROC curve. The area under the prognostic ROC curve (AUC) equals the probability that the time-to-event is shorter in high-risk patients than in low-risk patients^29^. The number of cases (n) and events (e) for low and high-expression groups is displayed. The *p*-value was calculated by the log-rank test.

In Kaplan–Meier analysis of the TMA_1 cohort, cyclin F-high expression was significantly associated with shortened median OS (34 months) compared with cyclin F-low (77 months; p = 0.003; Fig. 2a). As presented in Table 3, statistically significant univariable hazard ratios (HRs) were found for cyclin F (HR 1.91, 95% CI 1.23–2.98), age at diagnosis (HR 1.63, 95% CI 1.09–2.46), sex (HR 0.57, 95% CI 0.37–0.88), tumor grade (HR 3.46, 95% CI 1.90–6.31), and nodal status (HR 3.44, 95% CI 1.55–7.64). In a subsequent stepwise selection analysis, all these remained independent prognostic factors for OS (Table 3; all p < 0.05). The area under the prognostic ROC curve was 0.66: cyclin F overexpressors had a 66% probability of dying before cyclin F low expressors (Fig. 2b).

**Table 3:** Univariable and multivariable analyses of prognostic indicators by Cox regression model in the TMA_1 cohort.

Variable	n/EPV	Univariable analysis				Multivariable analysis^b^			
		HR	95% CI		*p*	HR	95% CI		*p*
			L	U			L	U	
Cyclin F (**high** vs low)	74/71_34/31	1.91	1.23	2.98	**0.004**	1.84	1.17	2.89	**0.009**
Age (**> 60** vs ≤ 60)	45/40_63/62	1.63	1.09	2.46	**0.019**	1.57	1.04	2.38	**0.033**
Sex (**male** vs female)	75/70_33/32	0.57	0.37	0.88	**0.010**	0.63	0.40	0.98	**0.039**
Tumor grade (**G3-G4** vs G1–G2)	13/13_95/89	3.46	1.90	6.31	**< 0.0001**	3.44	1.87	6.33	**0.0001**
pT status (**T3–T4** vs T1–T2)	45/41_63/61	1.71^a^	0.94	3.11	0.08	–	–	–	–
N status (**N +** vs N−)	7/7_101/95	3.44	1.55	7.64	**0.002**	3.07	1.37	6.86	**0.006**

^a^pT was added as a time-dependent variable.

^b^Final result of a multivariable Cox analysis with backward elimination of the nonsignificant covariates. The initial model included the following variables: cyclin F (C_p_ = 11), age, sex, tumor grade, pT status, and N status. HR is shown for the subgroup marked in bold.

*n* case number, *EPV* events per variable.

Univariable (Supplementary Fig. S4) and multivariable (Supplementary Table S2) comparisons of the H-score at the median cut-off showed similar associations with the OS.

**Figure 3:**
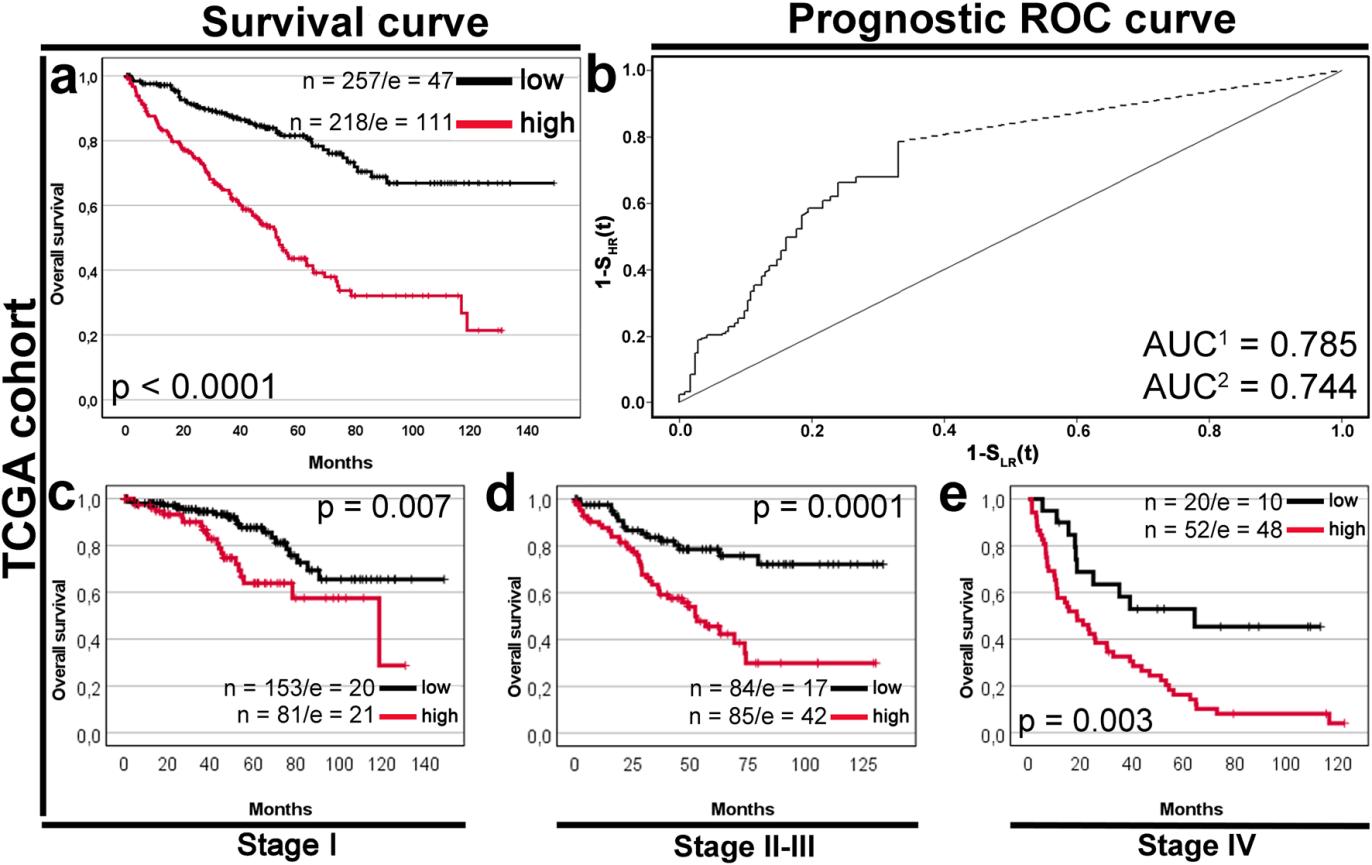
Effect of *CCNF* expression on survivals in The Cancer Genome Atlas (TCGA) data portal cohort. Cases were divided into low and high-expression groups according to the optimal cut-off point (C_p_ = 7.884) determined by the Evaluate Cutpoints software. (**a**) Kaplan–Meier curve comparing high and low expression of *CCNF* in the entire TCGA cohort (n = 475) and the corresponding (**b**) prognostic ROC curve. The area under the prognostic ROC curve (AUC) equals the probability that the time-to-event is shorter in high-risk patients than in low-risk patients. The missing part of the prognostic ROC curve (the straight dashed line) was extrapolated with optimistic (AUC^1^) and noninformative (AUC^2^) assumptions^29^. Kaplan–Meier curves comparing high and low expression of *CCNF* in (**c**) stage I subgroup (n = 234), (**d**) stage II–III subgroup (n = 169), and (**e**) stage IV subgroup (n = 72). *p*-values were calculated by the log-rank test. The number of cases (n) and events (e) in low and high expression groups is displayed.

In Kaplan–Meier survival analysis of the TCGA cohort, *CCNF*-high expression was significantly associated with a shorter OS in the whole cohort (52 months vs. not reached; p < 0.0001; Fig. 3a) as well as across all TNM stages (all p < 0.01; Fig. 3c–e). The areas under the prognostic ROC curve were 0.785 (an optimistic scenario) and 0.744 (a noninformative scenario) (Fig. 3b). In unadjusted Cox proportional hazards regression (Table 4), *CCNF* (HR 3.52, 95% CI 2.50–4.96), TNM stage (HR 3.61, 95% CI 2.59–5.02), T classification (HR 3.19, 95% CI 2.31–4.39), N classification (HR 3.78, 95% CI 2.01–7.13), and metastasis (HR 4.23, 95% CI 3.05–5.86) were significantly associated with OS. Importantly, multivariable Cox analysis demonstrated that *CCNF* remained a highly significant predictor of OS independently of histologic grade and TNM stage (HR 2.86, 95% CI 2.02–4.06, p < 0.0001; Table 4). Adjustment for pT, pN, and pM categories instead of AJCC disease stages yielded similar results for the *CCNF* expression (Supplementary Table S3). Further analysis through bioinformatics web resources, including GEPIA2, Kaplan–Meier Plotter, and GENT2 (Supplementary Fig. S5), confirmed that *CCNF* expression was highly discriminant for OS of ccRCC patients.

**Table 4:** Univariable and multivariable analyses of prognostic indicators by Cox regression model in the TCGA cohort.

Variable	n/EPV	Univariable analysis				Multivariable analysis			
		HR	95% CI		*p*	HR	95% CI		*p*
			L	U			L	U	
*CCNF* (**high** vs low)	218/111_257/47	3.52	2.50	4.96	**< 0.0001**	2.86	2.02	4.06	**< 0.0001**
Age (**> 60** vs ≤ 60)	236/83_239/75	1.06	0.77	1.44	0.74	–	–	–	–
Sex (**male** vs female)	312/101_163/57	0.95	0.68	1.31	0.74	–	–	–	–
Tumor grade (**G3–G4** vs G1–G2)	261/99_214/59	1.36	0.98	1.87	0.06	–	–	–	–
pT status (**T3–T4** vs T1–T2)	177/96_298/62	3.19	2.31	4.39	**< 0.0001**	–	–	–	–
pN−	225/78	Ref				–	–	–	–
**pN+**	15/11	3.78	2.01	7.13	**< 0.0001**	–	–	–	–
**pNx**	235/69	0.83	0.60	1.15	0.26	–	–	–	–
M status (**M1** vs M0)	69/56_406/102	4.23	3.05	5.86	**< 0.0001**	–	–	–	–
TNM stage (**III–IV** vs I–II)	191/105_284/53	3.61	2.59	5.02	**< 0.0001**	2.96	2.11	4.14	**< 0.0001**

*Ref.* reference.

The final result of a multivariable Cox analysis with backward elimination of the nonsignificant variables. The initial model included the following variables: *CCNF* (C_p_ = 7.884), tumor grade, and TNM stage. HR is shown for the subgroup marked in bold.

*n* case number, *EPV* events per variable.

### Association with tumor immune infiltration

**Figure 4:**
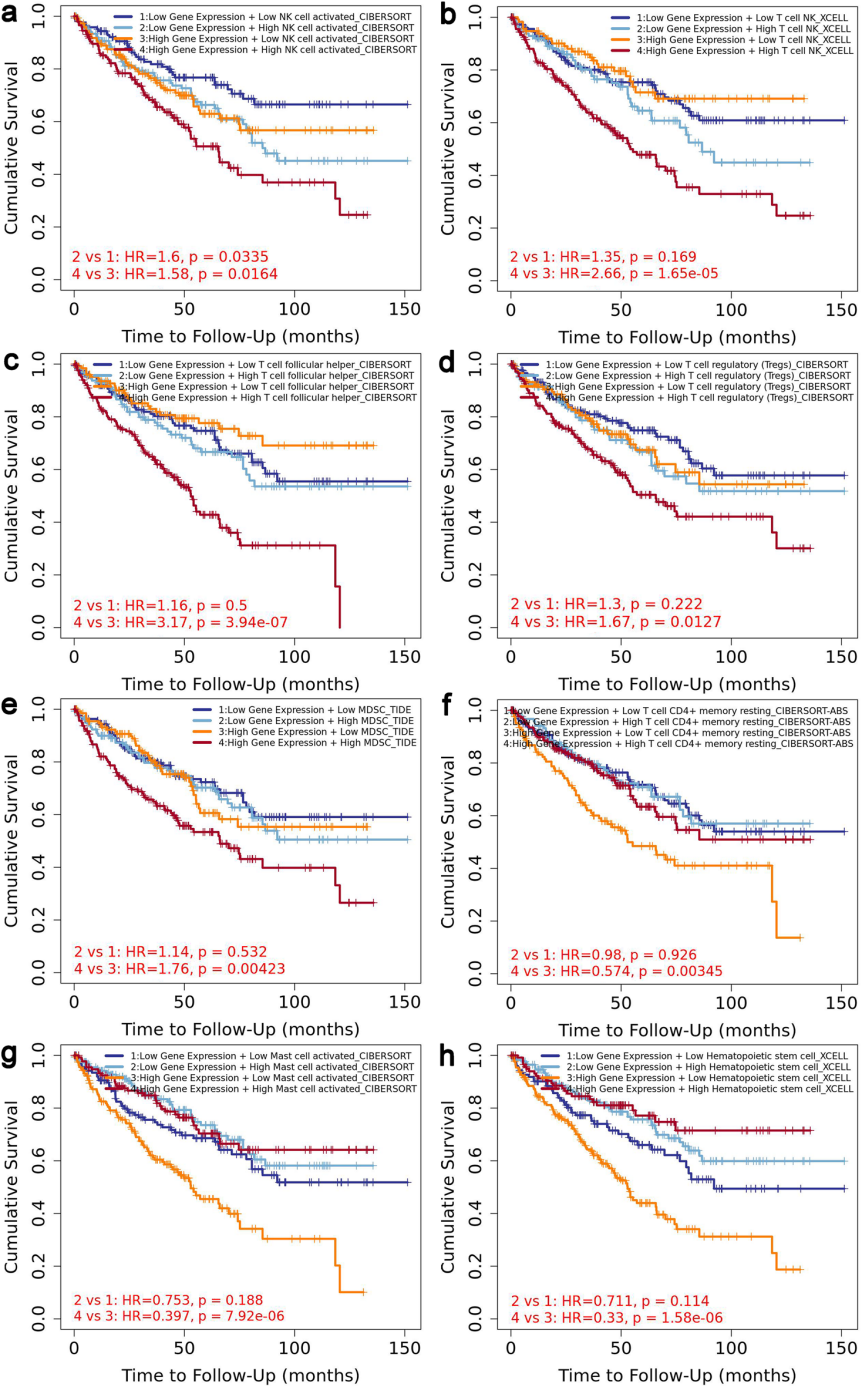
The association between survival and clear cell renal cell carcinoma defined by *CCNF* expression and immune infiltration level. Kaplan–Meier plots from the ‘Outcome Module’ of the Tumor IMmune Estimation Resource 2.0 (TIMER2) show the difference of overall survival among patients stratified by both *CCNF* expression level the estimated infiltration level of (**a**) activated NK cell; (**b**) T cell NK; (**c**) follicular helper T cells; (**d**) regulatory T (Tregs) cells; (**e**) myeloid-derived suppressor cells (MDSCs); (**f**) resting memory CD4 + T cells; (**g**) activated mast cells, and (**h**) hematopoietic stem cells (HSCs). The immune cell infiltration and *CCNF* expression were divided into high and low groups according to the median. The hazard ratio and the log-rank p value are shown.

Here, we investigated the potential of *CCNF* to display the prognostic significance of immune cells in KIRC. Using ‘Gene_DE’ and ‘Gene_Outcome’ modules of the TIMER2 web server, we first confirmed that *CCNF* was significantly upregulated in KIRC relative to adjacent normal kidney tissue and that *CCNF*-high expression reflected a poor clinical outcome in KIRC (Supplementary Fig. S6). Then, we demonstrated that *CCNF* expression had significant positive correlations with infiltrating levels of activated natural killer (NK) cells, T cell NK cells, follicular helper T (Tfh) cells, regulatory T (Tregs) cells, and myeloid-derived suppressor cells (MDSCs); and the patients with higher infiltration of these immune cells indicated an unfavorable prognosis (Supplementary Fig. S7). In turn, *CCNF* was negatively correlated with resting memory CD4 + T cells, activated mast cells, and hematopoietic stem cells (HSCs); and the patients with lower infiltration of these immune cells had an adverse prognosis (Supplementary Fig. S7). In addition, *CCNF* expression demonstrated a significant negative correlation with tumor purity in KIRC (r = − 0.122, p = 0.00853; data not shown). Importantly, KIRC patients with high *CCNF* expression and high NK cell infiltration had significantly worse prognosis than those with high *CCNF* expression and low infiltration of NK cells (HR = 1.58, p = 0.0164; Fig. 4a). For patients with low *CCNF* expression, high NK cell infiltration indicated poorer survival than those with lower infiltration level (HR = 1.6, p = 0.0335; Fig. 4a). Furthermore, KIRC patients with a particularly poor prognosis had both high expression of *CCNF* and increased infiltration degree of T cell NK cells (Fig. 4b), Tfh cells (Fig. 4c), Tregs (Fig. 4d), or MDSCs (Fig. 4e). Likewise, tumors with concomitant high expression of *CCNF* and lower infiltration of resting memory CD4 + T cells (Fig. 4f), activated mast cells (Fig. 4g), or HSCs (Fig. 4h) also experienced the worst prognosis.

Moreover, GEPIA2 was applied to explore the correlation between *CCNF* expression and gene markers of tumor-infiltrating immune cells in TCGA-KIRC. In particular*,* Treg-related gene signature had the strongest correlations with *CCNF* expression (r = 0.47, p < 0.0001; Supplementary Fig. S8). The results of GEPIA2 additionally showed the positive correlations between *CCNF* and TAM-associated markers, as well as M1 and M2 macrophage markers (Supplementary Fig. S8). We also determined the relationship between *CCNF* expression and T cell exhaustion-associated markers, including *HAVCR2* (*TIM-3*), *TIGIT*, *LAG3*, PD-1 *(PDCD1*), *CXCL13*, *LAYN*, *CTLA4*, *GZMB*. The results demonstrated that the *CCNF* expression level was positively correlated with the T*-*cell exhaustion signature in TCGA-KIRC (Supplementary Fig. S8).

### PPI network construction and functional enrichment analysis

**Figure 5:**
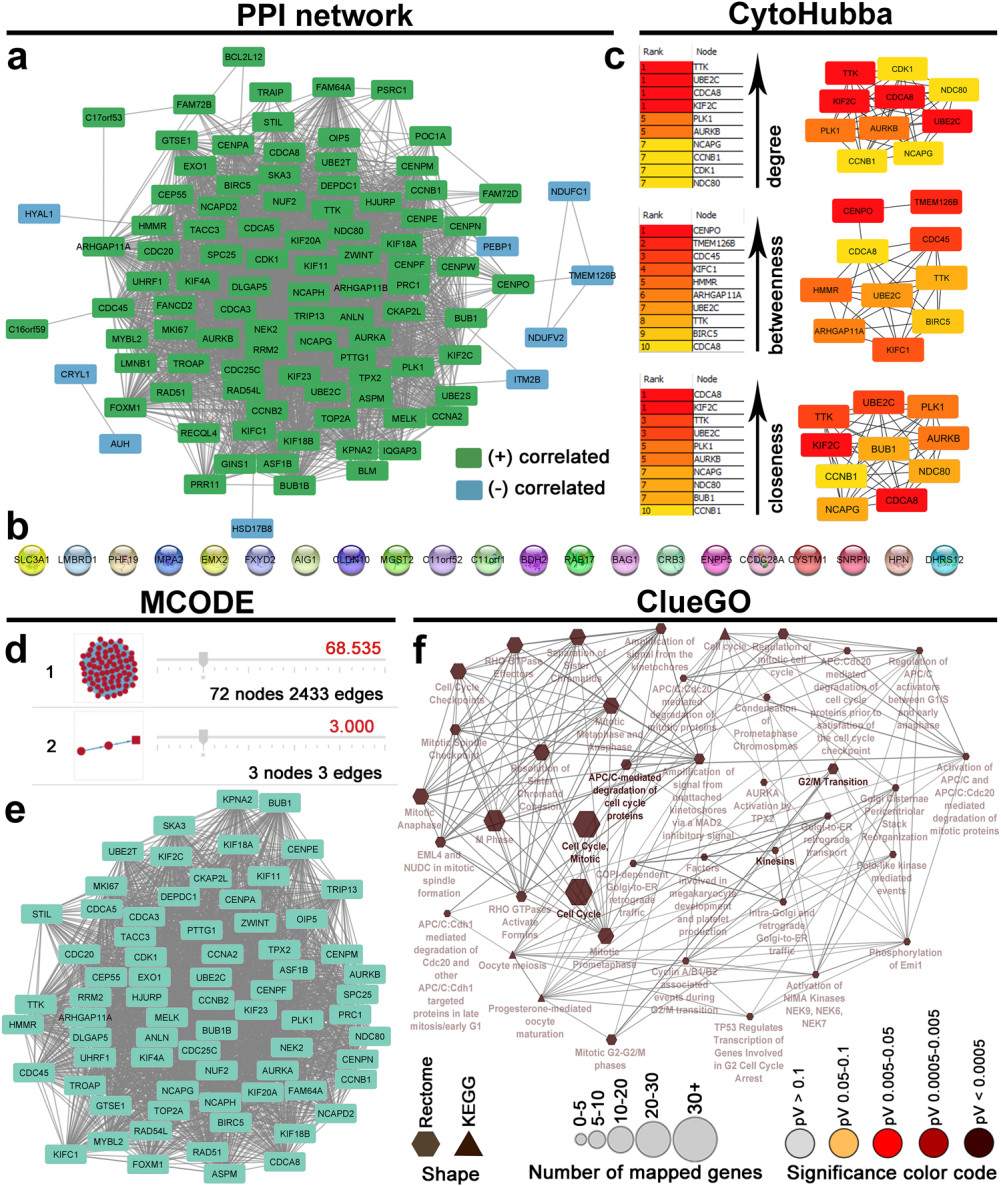
Functional enrichment analysis for *CCNF*-correlated genes. (**a**) Protein–protein interaction (PPI) network for the top genes positively (green nodes) or negatively (blue nodes) correlated with *CCNF* in clear cell renal cell carcinoma; (**b**) disconnected nodes; (**c**) The top 10 hub genes identified by CytoHubba Cytoscape plugin are ranked according to degree centrality, betweenness centrality, and closeness centrality; (**d**) The two modules identified in the PPI network using the MCODE Cytoscape plugin. (**e**) The top module (cluster 1) identified from the PPI network using the MCODE Cytoscape plugin; (**f**) Enrichment of nodes in the PPI network by Reactome Pathways and KEGG terms is illustrated using the ClueGO plugin.

Ninety-one genes positively correlated with *CCNF* and 30 genes negatively correlated with *CCNF* met the inclusion criteria*.* The PPI network consisted of a total of 100 nodes with 2917 edges (clustering coefficient 0.865, PPI enrichment p-value: < 1.0e−16), including 91 nodes positively correlated with *CCNF* and nine nodes negatively correlated with *CCNF* (Fig. 5a); the remaining nodes represented negatively correlated genes, which evidently were disconnected, as shown in Fig. 5b. The top 10 nodes with the highest degree, betweenness centrality, and closeness centrality are listed in Fig. 5c. The two clusters identified by MCODE are listed in Fig. 5d; this with a high clustering score (68.535) is shown in Fig. 5e. Functional enrichment analysis was then applied to the genes of cluster 1 to gain insights into the role they may play in ccRCC. The most significant (FDR-corrected p-value < 0.01) "Biological Process", "Cellular Components", and "Molecular functions" GO terms are listed in Supplementary Table S4. The enriched Reactome Pathways and KEGG terms related to the queried genes are depicted in Fig. 5f.

## Discussion

Cyclins, as master cell cycle regulators, and since their discovery in 1982, are in the spotlight of cancer research. Initially, cyclins were thought to be simple switches enabling cell cycle progression. Still, years of research have shown that their function is much more complex and can affect many cellular processes. For example, recent studies show the involvement of the cyclins and Cdks in molecular pathways associated with the regulation of transcription, epigenetic regulation, metabolism, stem cell self-renewal, neuronal functions, and spermatogenesis^30^. Increasing evidence also suggests the involvement of cyclin F/*CCNF* in cancer. Nevertheless, the data indicate that cyclin F is a multifaceted protein with an ambiguous role in cancer cells. In this study, we investigated the prognostic value of cyclin F/*CCNF* in ccRCC patients.

As far as we know, prior to our study, the prognostic value of cyclin F protein had not yet been investigated in the context of ccRCC. However, *CCNF* gene expression has recently been reported as a promising biomarker for prognosis prediction in ccRCC^31–34^. Here, by integrating analyses through IHC, RNA-seq, and gene chip microarray data, we reproducibly found the remarkable differences in CCNF protein and gene expression levels between tumor and control tissues. Furthermore, cyclin F immunoreactivity was observed in most ccRCCs, in a minority of tumor-adjacent normal tissues, and neither of normal renal tissues. Moreover, tumors with features of more aggressive behavior were more likely to display cyclin F-high expression than low. These results suggest that cyclin F overexpression may provide a selective advantage in ccRCC pathobiology. Furthermore, they further support the notion that normal adjacent to tumor tissue represents a unique intermediate state between healthy and tumor tissue^35^. Finally, they also highlight the attractiveness of cyclin F as a biomarker in ccRCC, as the on–off expression pattern facilitates the IHC staining interpretation in routine practice^36^.

Previous studies have shown that altered *CCNF* expression is associated with a poor prognosis in various cancers, including breast, liver, pancreatic, brain, and skin cancers^12–14, 37^. In the current study, the analysis of the data from the TCGA database reveals that 45.89% of ccRCC patients are characterized by high *CCNF* mRNA expression. Furthermore, overexpression of *CCNF* in these individuals is associated with a poorer prognosis, advanced T and TNM stage, and distant as well as lymph node metastasis. Moreover, the expression of *CCNF* increases proportionally with tumor grade. Indeed, Wei et al. have recently shown that the mRNA expression levels of *CCNF* in KIRC-TCGA increased gradually with a disease progression and correlated with adverse OS and RFS^31^. These observations are also in agreement with some other studies investigating *CCNF* mRNA levels in different tumors. Our former analysis on melanoma and pancreatic adenocarcinoma also revealed that high *CCNF* mRNA level was associated with a worse prognosis for the patients^12,37^. Similarly, the study presented by Liu and colleagues demonstrated a strong relationship between high *CCNF* mRNA levels and worse prognosis in breast cancer (BC) patients. Moreover, *CCNF* overexpression significantly correlated with worse RFS, OS, and DMFS among all types^38^. Importantly, these observations have been confirmed on the protein level where high expression of cyclin F was observed in Luminal A, Luminal B, HER2, and TNBC breast cancer. Additionally, the in vitro experiments showed that silencing cyclin F reduces proliferation rate and migration of breast cancer cells^38^. Another example is liver cancer, where an elevated level of *CCNF* was associated with worse OS. Additionally, this overexpression cannot be led by the mutations in the *CCNF* gene because only 0.81% of 372 liver cancer patients bear the mutation in *CCNF* locus^14^.

Interestingly, the IHC study by Fu et al. on in-house liver cancer series showed the opposite, including the association of cyclin F-low with more aggressive tumor features and a prognosis^11^. The liver cancer studies mentioned above^11,14^ present the opposite prognostic value of cyclin F depending on the source of data or, more precisely, the measurement level (protein vs. mRNA), leading to further controversy regarding the functional portrait of this cyclin in human cancers. We witnessed similar discrepancies in our studies on melanoma and pancreatic adenocarcinoma. The bioinformatics analysis of melanoma patients pointed to *CCNF* as a negative prognostic marker, and it was reasonable to consider *CCNF* as an oncogene. However, the in vitro studies do not confirm the assumption that *CCNF* is an oncogene. After cyclin F silencing, we observed an increased proliferation rate and elevated capabilities for migration and invasion. Additionally, the morphological and molecular alterations suggested the induction of the process similar to epithelial-to-mesenchymal transition in the A375 melanoma cell line^12,39^. In pancreatic adenocarcinoma, high *CCNF* mRNA was associated with poor prognosis, whereas the univariable Cox proportional hazards model pointed to cyclin F protein as a favorable prognostic marker; however, the last observation was not statistically significant^37^. Here, we obtained consistent results from both TCGA and TMA cohorts. TCGA and TMA_1 cohorts are characterized by reduced OS in cyclin F-high groups. Furthermore, in TMA and TCGA cohorts, patients with more aggressive tumor features more frequently had cyclin F/*CCNF*-high than cyclin F/*CCNF*-low expression. All presented data suggest a potential oncogenic role for cyclin F in clear cell renal cell cancer . By contrast, Deshmukh et al. presented the oncosuppressive properties of cyclin F in glioma xenografts. The authors observed a decrease in cyclin F expression simultaneously with the increase in tumor grade^40^.

To shed more light on the underlying mechanisms behind the prognostic value of *CCNF* expression in ccRCC, we identified *CCNF*-correlated genes, and then built PPI network and performed functional enrichment analyses of the most significant gene cluster. All of the nodes of cluster 1 genes are highly interacting and therefore act as hub (high degree) genes in the PPI network, and may be corresponding to the ccRCC-causing genes^41^. As can be seen, the vast majority of the hub genes identified in the PPI by calculating centrality parameters (using the CytoHubba plugin) are also present in the cluster 1, which futher emphasizes their role in cancer-relevant pathways. Generally, cluster 1 showed the enrichment for gene sets associated with cell cycle and cell division, in particular M phase and mitotic checkpoint, being highly relevant to the control of cell growth and proliferation. In this context, it is essential that overexpression of *CCNF* is positively correlated with proliferation-promoting genes, suggesting that it contributes to cancer progression and therefore poor patient prognosis by promoting excessive cell proliferation*.* Indeed, many of our hub genes are already known from their ocogenic and pro-proliferative activities in ccRCC*.* The node TTK possesses the highest degree centrality and has been shown to play an important role in maintaining the proliferative and invasive potential of ccRCC cells, both in vitro and in vivo in mice^42^. In our PPI network, *TTK* interacts with *UBE2C*^43^, *CDCA8*^44^, *KIF2C*^45^, *PLK1*^46^, *AURKB*^47^, *NCAPG*^48^, *CCNB1*^49^, *CDK1*^48^, *NDC80*^50^, all of which have been previously implicated in the initiation and/or progression of ccRCC. Furthermore, many of the upstream or downstream regulators of the listed genes may activate deleterious signaling (e.g. phosphorylation) pathways, having established connections with human cancers, including ccRCC. Also notably, these highly-related genes in the PPI network may reciprocally control each other or be affected by the same transcription factors, thus targeting one may interrupt the entire cluster and even the whole PPI network. For example, *UBE2C*, which is detected as the second pivotal hub gene in the present study, is a transcriptional target of *FOXM1*, which is also included in cluster 1*.* On the other hand, transcriptional activity of FOXM1 is strongly dependent on phosphorylation by PLK, which therefore controls the execution of the transcriptional program required for mitotic progression of ccRCC cells^46^. FOXM1 is proven to be one of the key transcription factors regulating oncogenic signalling pathways during the ccRCC progression. Together with UBE2C, it activates AKT/mTOR signaling pathway to increase the cell cycle progression, proliferation rate, cell survival, as well as the migratory and invasive abilities of ccRCC cells^51^. In addition, FOXM1/KIF20A axis executes oncogenic activity in ccRCC via activation of the EMT signaling^52^, while FOXM1/AURKB axis via stimulation of cancer cell proliferation^51^. Importanlty, both *KIF20A* and *AURKB* are included in cluster 1 genes. AURKB decreases the expression of the cell cycle inhibitor P21 by inhibiting P53 activity, subsequently causing upregulation of CDK1, eventually leading to cell division and increasing the tumor cell survival^53^. CDK1 is also affected by the proportion of FOXM1 and KIF20A overexpression^54^, which further highlights their decisive roles in the cell division and mitosis. In fact, our functional analyses are consistent with recent in silico and in vitro studies of Li et al., who showed that *CCNF* might promote ccRCC proliferation by inhibiting cell senescence through the CDK1-P53 signaling pathway^33^. Taken together, it appears that *CCNF* and its highly correlated genes initiate the signaling pathways that eventually result in uncontrolled cell proliferation, thus targeting the cyclin F offers a potential novel strategy to target the ccRCC cells efficiently.

Another aspect of this study was to estimate the relevance between the *CCNF* and tumor immune infiltration in TCGA-KIRC. In agreement with the recent studies of Gao et al.^32^, as well as Wei et al.^31^, our analysis showed that *CCNF* was closely associated with immune cell infiltration in TCGA-KIRC. Interestingly, *CCNF* was positively correlated with the infiltration abundance of activated NK cells, TNK cells, Tfh cells, Tregs, and MDSCs, the high expression of which predicted poor survival in KIRC-TCGA patients, while it was negatively correlated with resting memory CD4 + T cells, activated mast cells, and HSCs that conferred a better prognosis when present in tumor microenvironment*.* Furthermore, here we also explored the difference in overall survival among KIRC patients stratified by both the estimated infiltration level of immune cells and *CCNF* expression level. Specifically, we found that KIRC patients with a particularly poor prognosis had concomitantly high expression of *CCNF* and increased infiltration of NK cells, TNK cells, Tfh cells, Tregs, MDSCs, or decreased abundance of resting memory CD4 + T cells, activated mast cells, or HSCs. These results suggest that immune cell infiltration may be one of the reasons that caused *CCNF* to become a prognostic factor in ccRCC. Furthermore, in addition to having the strongest correlations with Treg-related gene signature, *CCNF* expression was also highly significantly (p < 0.0001) associated with gene markers of, for example, the M2 macrophages and exhausted T cells. We can therefore speculate that *CCNF* is involved in immune escape and immunosuppression in the ccRCC microenvironment, and it may serve as a potential target to increase the effectiveness of immunotherapy in ccRCC. However, the abundance and gene markers of different tumor-infiltrating immune cells were found to be only weakly to moderately correlated with *CCNF* (the range for correlation coefficients: 0.1–0.47). This is probably why, we did not find *CCNF*-correlated genes to be highly enriched in immune processes and immune-related pathways via our functional enrichment analysis, which was set at high confidence.

Our study has some limitations that weaken the conclusiveness of the presented data. First, it is impossible to directly correlate the protein data from our cohort and the RNA-seq data from the TCGA cohort. Simultaneously analyzing mRNA and protein from the same sample would significantly improve the quality of the conclusions by providing a more comprehensive understanding of the relationship between mRNA expression and protein levels. Also, the structure of the cohorts differs from each other. For example, in the TMA_1 cohort, 65.74% of the patients were diagnosed with grade 2 ccRCC. On the other hand, in TMA_2, 80.23% were diagnosed with grade 1 ccRCC, whereas in the TCGA cohort, 54.95% were diagnosed with grades 3–4. Moreover, although the present study goes some way towards understanding the potential mechanisms underlying how *CCNF* affects ccRCC, it is clear that our bioinformatics analyses still need further verification through in vivo and in vitro experiments. Notwithstanding the described limitations, our study provides strong evidence for the utility of cyclin F as a prognostic and diagnostic marker in ccRCC.

In conclusion, by analyzing protein levels and publicly available transcriptomic data, we found that cyclin F/*CCNF* is significantly upregulated in ccRCC patients. High expression of cyclin F/*CCNF* is an unfavorable prognostic factor that correlates with aggressive tumor phenotype and poor patient survival. Mechanistically, our bioinformatics analyses suggest that *CCNF* may act as an oncogene in ccRCC via promoting cancer cell proliferation and affecting the tumor immune microenvironment. Further investigation, including larger cohort and in vitro studies, is needed to describe molecular pathways altered along with *CCNF* overexpression.

### Supplementary Information

Supplementary Information.

## Data Availability

Publicly available datasets were analyzed in this study. These data can be found here: https://www.cbioportal.org (accessed on 14 September 2022); https://xenabrowser.net/, (accessed on 14 September 2022); http://tnmplot.com (accessed on 28 November 2022); http://gdancik.github.io/shinyGEO/ (accessed on 30 November 2022); http://ualcan.path.uab.edu/ (accessed on 30 November 2022 and 23 March 2023); http://gent2.appex.kr/gent2/ (accessed on 29 November 2022); http://gepia2.cancer-pku.cn/ (accessed on 13 December 2022); http://kmplot.com/analysis (accessed on 13 December 2022); https://david.ncifcrf.gov (accessed on 23 March 2023). Own TMA_1 dataset used and analyzed during the current study are available from the corresponding author on reasonable request. A commercially purchased TMA_2 dataset is available online at https://www.tissuearray.com/ under the no. KD20810a.
