# Supplementary material for: Overexpression of cyclin F/CCNF as an independent prognostic factor for poor survival in clear cell renal cell carcinoma
Source: Sci Rep. 2024 Apr 23;14:9280. doi: 10.1038/s41598-024-59437-1 (PMC11039610; doi:10.1038/s41598-024-59437-1)
Supplement: Supplementary file 1 — Supplementary Information. [file 41598_2024_59437_MOESM1_ESM.pdf]

Overexpression of cyclin F/*CCNF* as an independent prognostic factor for poor survival in clear cell renal cell carcinoma

Maciej Kwiatkowski, Adrian Krajewski, Justyna Durślewicz, Karolina Buchholz, Dariusz Grzanka, Maciej Gagat, Jan Zabrzyński, Anna Klimaszewska-Wiśniewska

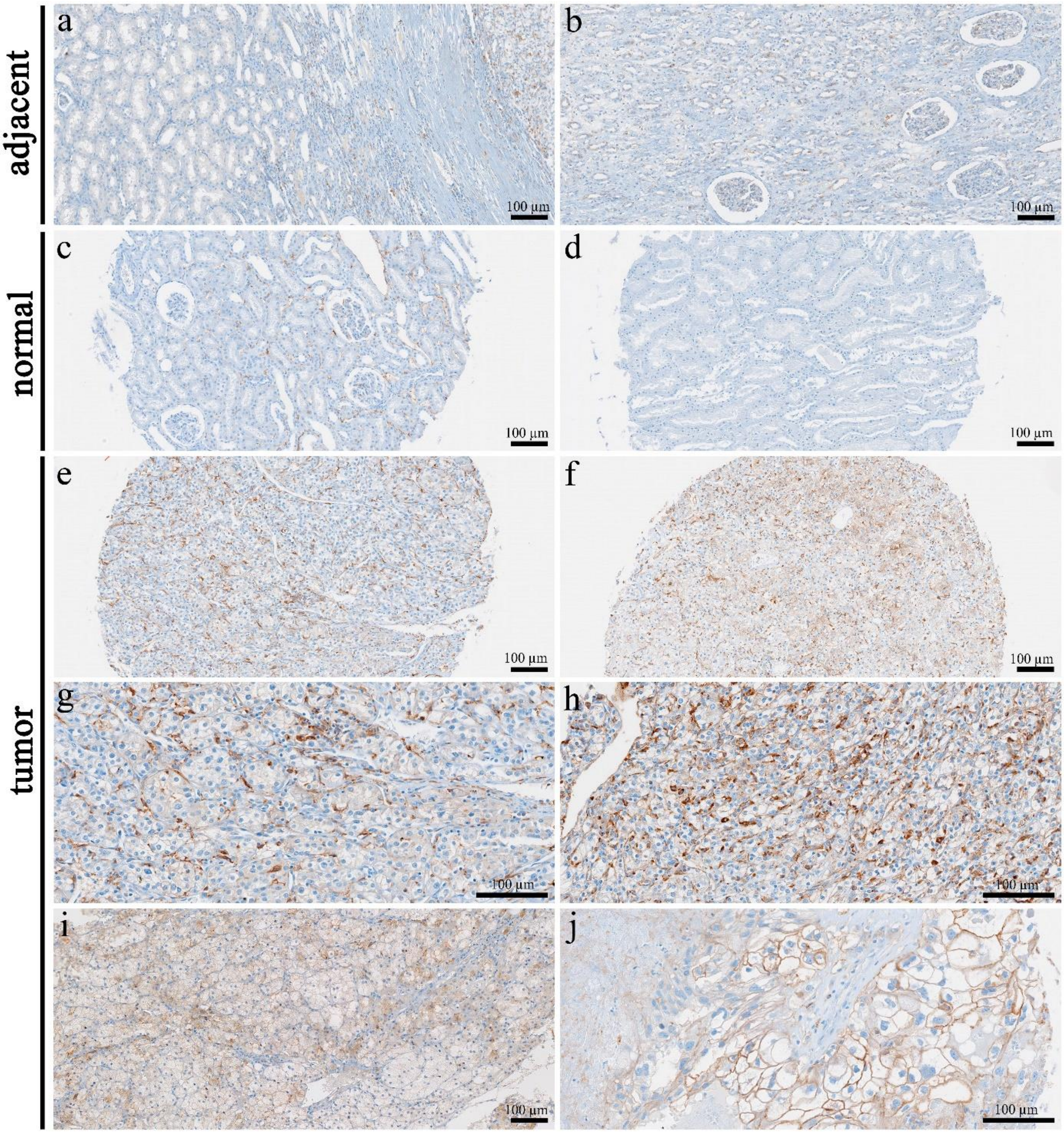

Figure S1. Representative photographs showing immunohistochemical expression of cyclin F in clear cell renal cell carcinoma and control tissues. (a, b) Tumor-adjacent normal tissue; (c, d) normal renal tissue; (e-j) tumor tissue from (a, b, g-i) TMA_1 and (c-f, j) TMA_2 cohorts; a-f and i: original magnifications: 10×, g, h, j: original magnifications: 20×. Bar 100 µm.

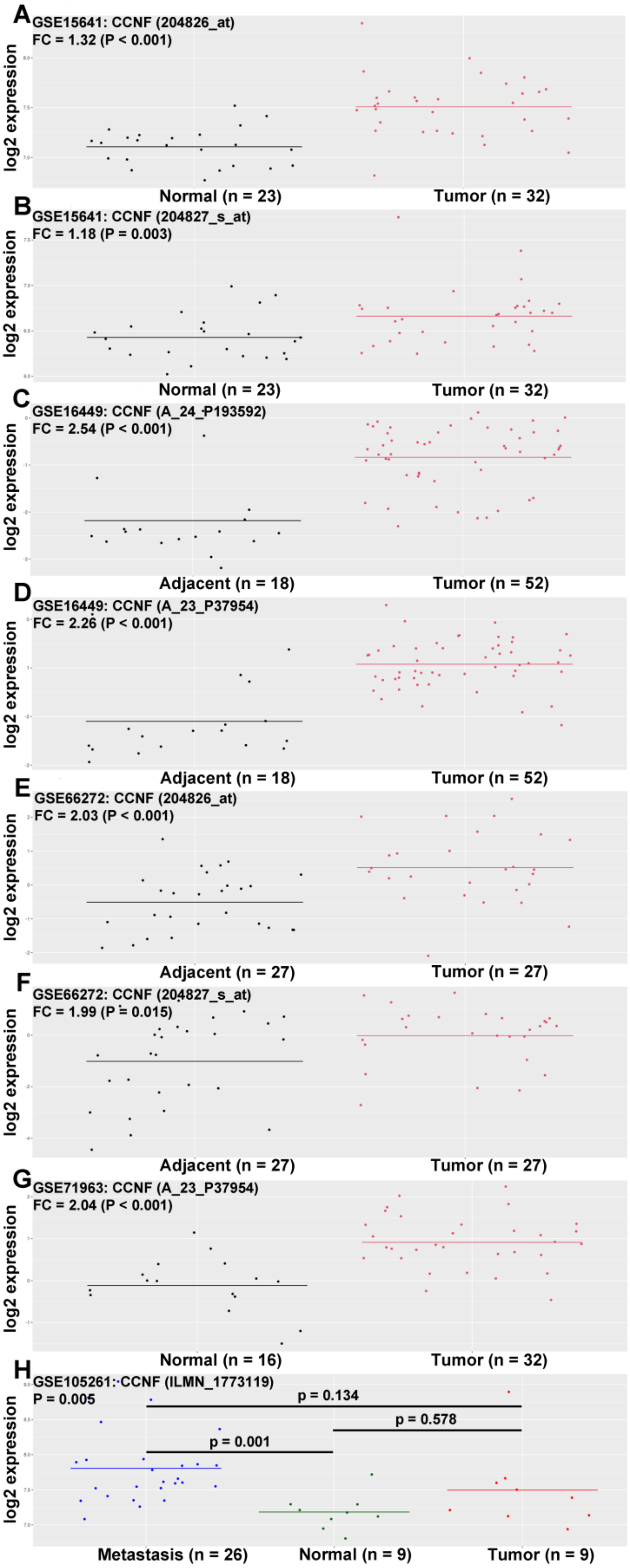
A
GSE15641: CCNF (204826_at)
FC = 1.32 (P < 0.001)
log2 expression
Normal (n = 23)
Tumor (n = 32)
B
GSE15641: CCNF (204827_s_at)
FC = 1.18 (P = 0.003)
log2 expression
Normal (n = 23)
Tumor (n = 32)
C
GSE16449: CCNF (A_24_P193592)
FC = 2.54 (P < 0.001)
log2 expression
Adjacent (n = 18)
Tumor (n = 52)
D
GSE16449: CCNF (A_23_P37954)
FC = 2.26 (P < 0.001)
log2 expression
Adjacent (n = 18)
Tumor (n = 52)
E
GSE66272: CCNF (204826_at)
FC = 2.03 (P < 0.001)
log2 expression
Adjacent (n = 27)
Tumor (n = 27)
F
GSE66272: CCNF (204827_s_at)
FC = 1.99 (P = 0.015)
log2 expression
Adjacent (n = 27)
Tumor (n = 27)
G
GSE71963: CCNF (A_23_P37954)
FC = 2.04 (P < 0.001)
log2 expression
Normal (n = 16)
Tumor (n = 32)
H
GSE105261: CCNF (ILMN_1773119)
P = 0.005
p = 0.134
p = 0.578
p = 0.001
log2 expression
Metastasis (n = 26)
Normal (n = 9)
Tumor (n = 9)

Figure S2. Expression of *CCNF* in clear cell renal cell carcinoma compared to (a-h) non-cancerous control tissue and/or (h) metastasis based on the ShinyGEO web portal**.** Information about the study name, case numbers, GSE, and probe data is shown. Fold change (FC) represents the expression of the *CCNF* in a tumor relative to that in healthy controls. When appropriate, P-values were calculated by the Mann-Whitney or Wilcoxon test or Kruskal–Wallis test with Dunn's post hoc test.

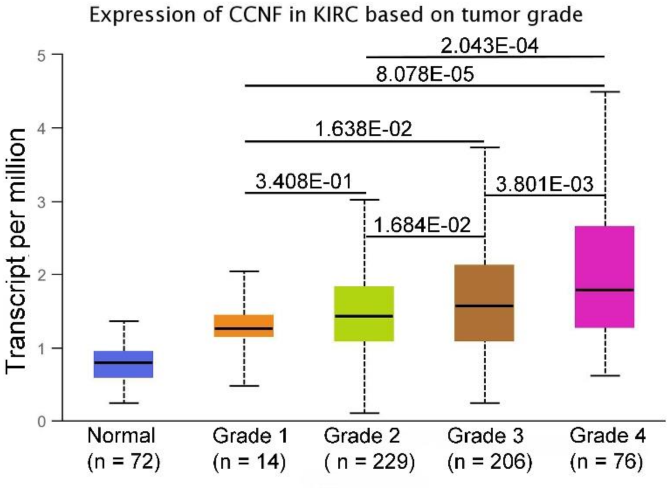

Figure S3. Association between the *CCNF* expression level and tumor grade based on the TCGA data via the UALCAN web portal. The box plot shows the relative expression of *CCNF* in normal and kidney renal clear cell carcinoma (KIRC) samples classified by tumor grade. Statistical significance was assessed by the Student's *t*-test (unequal variance).

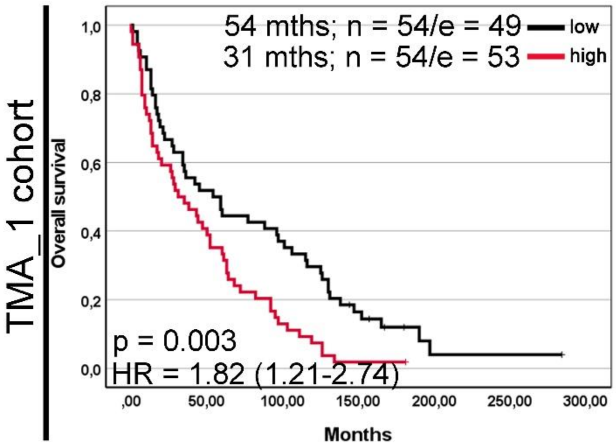

Figure S4. Effect of cyclin F expression on survivals in clear cell renal cell carcinoma cohort. Cases were divided into low and high-expression groups according to the median (C_p = 21.5). Kaplan-Meier curve comparing high and low expression of cyclin F in the entire TMA_1 cohort (n = 108). Median overall survival (in months; abbr. mths) for high and low expression groups and the p-value from the log-rank test and hazard ratio (HR) from the Cox model with a 95% confidence interval have been indicated.

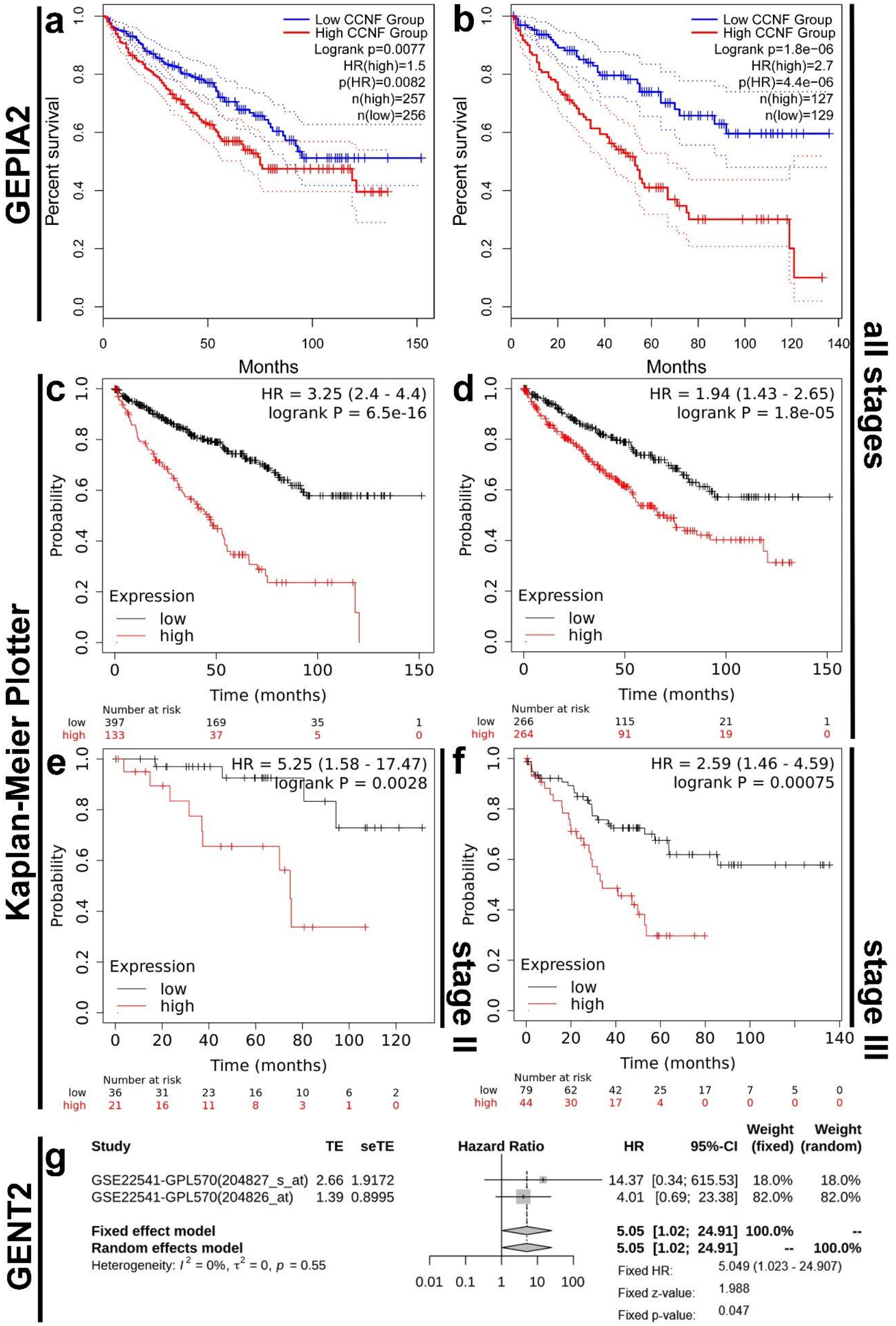

Figure S5. Effect of *CCNF* expression on the overall survival in clear cell renal cell carcinoma using the (a, b) GEPIA2, (c-f) Kaplan-Meier Plotter, and (g) GENT2 web portals. Cases were divided into low and high-expression groups according to the (c, e, f) optimal cut-off point, (a, d) median, or (b) quartile expression. (a-d) Kaplan-Meier curves for *CCNF* expression in all tumor stages; (e) Kaplan-Meier curve for *CCNF* expression in stage II tumors; (e) Kaplan-Meier curve for *CCNF* expression in stage III

tumors. *P*-values were calculated by the log-rank test. (g) Forest plot of hazard ratio on the *CCNF* gene with the overall survival. The study name, GSE, GPL, and probe information are depicted. TE is the estimated treatment effect, seTE is the standard error of the treatment estimate, and HR indicates the hazard ratio average. Fixed and random effects are presented.

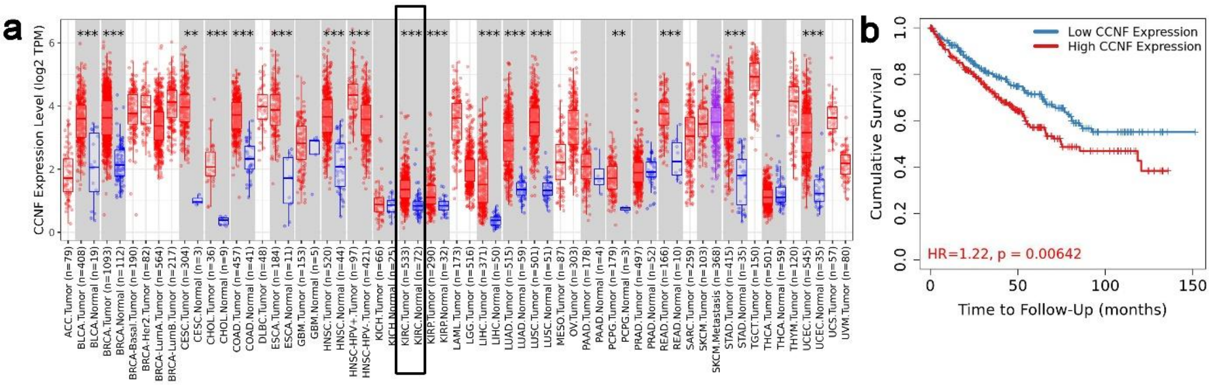

Figure S6. *CCNF* expression and association with survival in kidney renal clear cell carcinoma (KIRC) using the Tumor IMmune Estimation Resource 2.0 (TIMER2). (a) Box plots from the ‘Gene_DE’ module of TIMER2 show the differential expression between tumor and adjacent normal tissues for *CCNF* across all TCGA tumors (the results for KIRC are boxed). The statistical significance computed by the Wilcoxon test is annotated by the number of stars (*: p-value < 0.05; **: p-value <0.01; ***: p-value <0.001); (b) Kaplan–Meier plot from the ‘Outcome_Module’ of the TIMER2 shows the difference of overall survival among patients stratified by *CCNF* expression (high versus low at the median). The hazard ratio and the log-rank p value are shown.

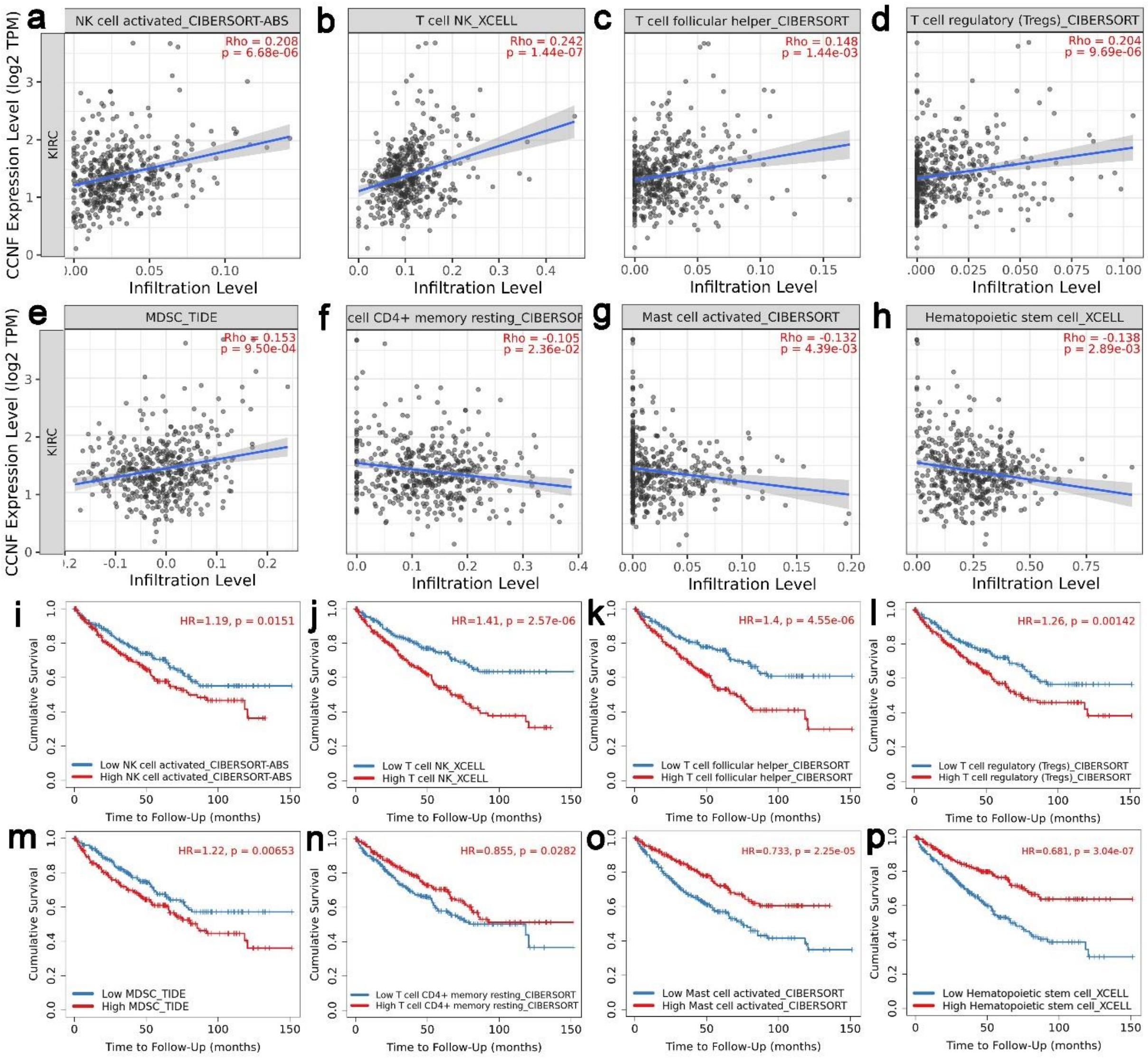

Figure S7. Immune infiltration level and survival time in clear cell renal cell carcinoma based on the Tumor IMmune Estimation Resource 2.0 (TIMER2). Correlation of *CCNF* expression with infiltrating levels of (a) activated NK cells; (b) T cell NK cells; (c) follicular helper T cells; (d) regulatory T (Tregs) cells; (e) myeloid-derived suppressor cells (MDSCs); (f) resting memory CD4+ T cells; (g) activated mast cells; (h) and hematopoietic stem cells. The relationships between overall survival and (i) activated NK cells; (j) T cell NK cells; (k) follicular helper T cells; (l) regulatory T (Tregs) cells; (m) myeloid-derived suppressor cells (MDSCs); (n) resting memory CD4+ T cells; (o) activated mast cells; (p) and hematopoietic stem cells. The immune cell infiltration was divided into high and low groups according to the median. The hazard ratio and the log-rank p value are shown.

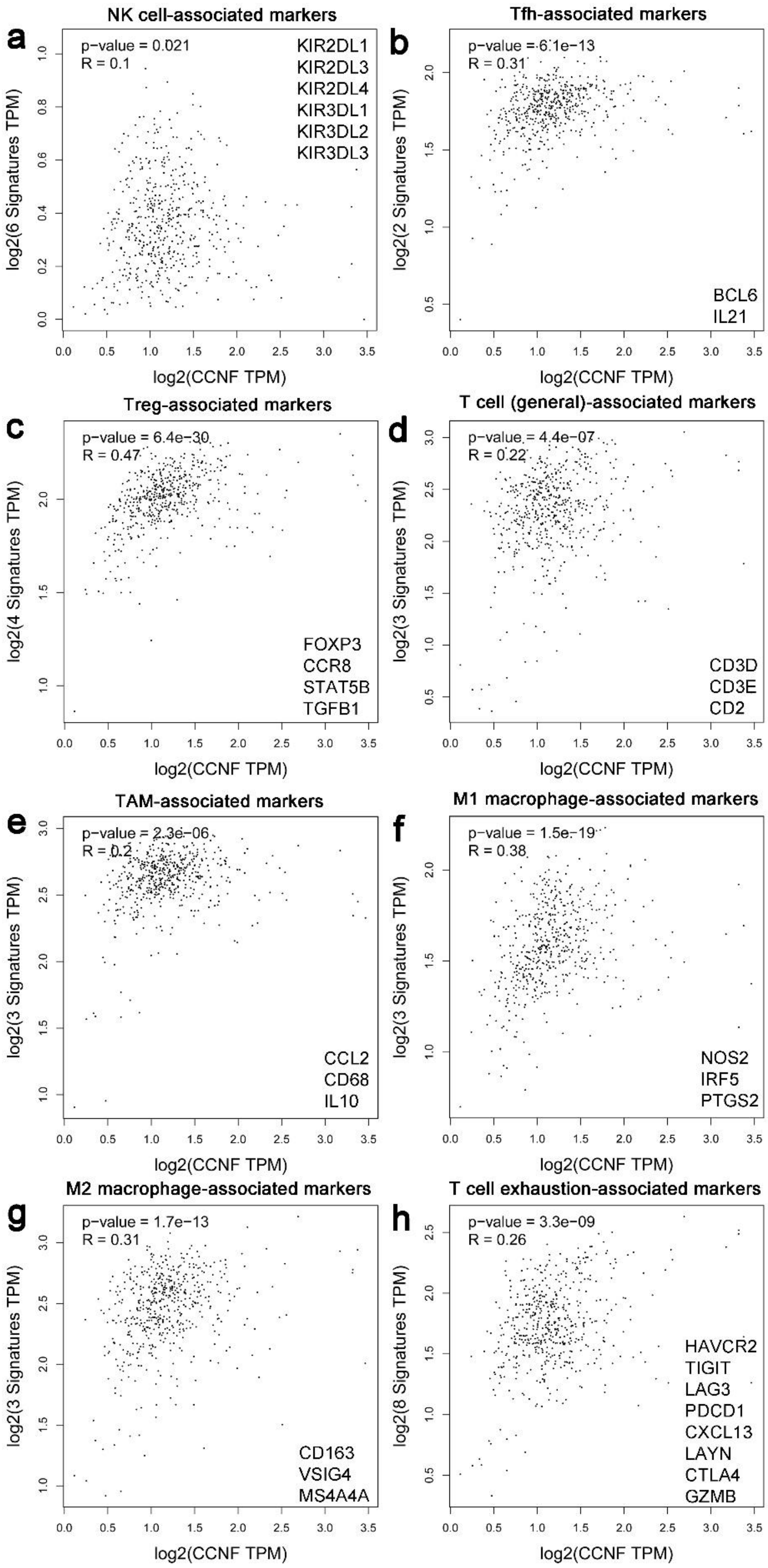

Figure S8. Correlation analysis between *CCNF* expression and gene markers of tumor-infiltrating immune cells in the GEPIA2 database. The scatter plots show the correlation of *CCNF* expression and the markers of (a) NK cells; (b) follicular helper T (Tfh) cells; (c) regulatory T (Tregs) cells; (d) T cells; (e) tumor associated macrophages (TAMs); (f) M1 macrophages; (g) M2 macrophages; (h) T cell exhaustion. Spearman's rho value, p-value, and immune infiltration associated markers are shown.

Table S1. Baseline characteristics of TMA_1 (n = 108), TMA_2 (n = 192), and TCGA (n = 475) patient cohorts.

Variable	TMA_1 cohort (%)	TMA_2 cohort (%)	TCGA cohort (%)
Age at diagnosis			
≤ 60	45 (41.67)	118 (61.46)	239 (50.32)
> 60	63 (58.33)	74 (38.54)	236 (49.68)
Mean ± SD	62.81±10.00	56.78 ±11.99	60.69 ±11.96
Sex			
Male	75 (69.44)	132 (68.75)	312 (65.68)
Female	33 (30.56)	60 (31.25)	163 (34.32)
Tumor grade			
Gx	0 (0.00)	12 (6,25)	0 (0.00)
G1	24 (22.22)	150 (78.13)	11 (2.32)
G2	71 (65.74)	29 (15.10)	203 (42.74)
G3	12 (11.11)	1 (0.52)	189 (39.79)
G4	1 (0.93)	0 (0.00)	72 (15.16)
pT status			
T1	34 (31.48)	133 (69.27)	237 (49.89)
T2	29 (26.85)	53 (27.60)	61 (12.84)
T3	42 (38.89)	5 (2.60)	167 (35.16)
T4	3 (2.78)	1 (0.52)	10 (2.11)
N status			
Nx	0 (0.00)	0 (0.00)	235 (49.47)
N0	101 (93.52)	191 (99.48)	225 (47.37)
N1	7 (6.48)	1 (0.52)	15 (3.15)
pM status			
Mx	-	0 (0.00)	15 (3.16)
M0	-	191 (99.48)	391 (82.32)
M1	-	1 (0.52)	69 (14.52)
TNM stage			
I	-	134 (69.79)	234 (49.26)
II	-	52 (27.08)	50 (10.53)
III	-	5 (2.60)	119 (25.05)
IV	-	1 (0.52)	72 (15.15)
OS status			
Alive	6 (5.55)	-	317 (66.74)
dead	102 (94.44)	-	158 (33.26)

SD, standard deviation, OS, overall survial

Table S2. Multivariable analysis of prognostic indicators by Cox regression model in TMA_1 cohort with the median H-score as the cut-off value for cyclin F expression.

Variable	n/EPV	Multivariable model			
		HR	95% CI		***p***
			L	U	
Cyclin F (**high** vs low)	54/53_54/49	1.93	1.25	2.99	**0.003**
Age (**>60** vs ≤60)	45/40_63/62	1.66	1.09	2.52	**0.018**
Sex **(male** vs female)	75/70_33/32	0.65	0.42	1.01	0.056
Tumor grade (**G3-G4** vs G1-G2)	13/13_95/89	3.93	2.12	7.29	**<0.0001**
pT (**T3-T4** vs T1-T2)*	45/41_63/61	-	-	-	-
N status (**N+** vs N-)	7/7_101/95	2.73	1.21	6.15	**0.015**

* pT was added as a time-dependent variable.

# Final result of a multivariable Cox analysis with backward elimination of the nonsignificant covariates. The initial model included the following variables: cyclin F (C_p = 21.5), age, sex, tumor grade, pT status, and N status. HR is shown for the subgroup marked in bold.

Table S3. Multivariable analysis of prognostic indicators by the Cox regression model in the TCGA cohort with pT, pN, and pM categories as the adjusting variables instead of the AJCC TNM stages.

Variable	n/EPV	Multivariable model			
		HR	95% CI		*p*
			L	U	
CCNF (**high** vs low)	218/111_257/47	2.72	1.90	3.90	**<0.0001**
Age (**>60** vs ≤60)	236/83_239/75	-	-	-	-
Sex **(male** vs female)	312/101_163/57	-	-	-	-
Tumor grade (**G3-G4** vs G1-G2)	261/99_214/59	-	-	-	-
pT status (**T3-T4** vs T1-T2)	177/96_298/62	1.81	1.25	2.61	**0.002**
pN-	225/78		Ref.		
**pN+**	15/11	1.93	1.01	3.70	**0.047**
**pNx**	235/69	0.69	0.50	0.97	**0.02**
M status (**M1** vs M0)	69/56_406/102	2.52	1.73	3.66	**<0.0001**

Ref. reference
The result of a multivariable Cox analysis with backward elimination of the nonsignificant variables. The initial models included the following variables: *CCNF* (Cp = 7.884), tumor grade, pT, pN, and M. HR is shown for the subgroup marked in bold.

Table S4. Gene ontology (GO) enrichment analysis for the cluster 1.

GO TERMS	GO ID	COUNT	FDR P-VALUE
**BIOLOGICAL PROCESSES (BPs)**			
Cell division	GO:0051301	36	5.84E-36
mitotic spindle organization	GO:0007052	16	1.40E-22
mitotic spindle assembly checkpoint	GO:0007094	13	4.17E-20
chromosome segregation	GO:0007059	14	5.51E-17
mitotic cell cycle	GO:0000278	16	4.76E-16
mitotic sister chromatid segregation	GO:0000070	9	2.38E-11
mitotic cytokinesis	GO:0000281	10	1.07E-10
G2/M transition of mitotic cell cycle	GO:0000086	9	1.81E-09
mitotic spindle midzone assembly	GO:0051256	6	9.54E-09
Microtubule-based movement	GO:0007018	9	3.68E-08
cell cycle	GO:0007049	13	1.80E-07
mitotic metaphase plate congression	GO:0007080	7	2.47E-07
kinetochore assembly	GO:0051382	5	5.72E-06
mitotic spindle assembly	GO:0090307	6	8.60E-06
spindle organization	GO:0007051	5	8.60E-06
mitotic chromosome condensation	GO:0007076	5	1.03E-05
regulation of chromosome segregation	GO:0051983	4	1.86E-04

positive regulation of mitotic cell cycle spindle assembly checkpoint	GO:0090267	4	1.86E-04
attachment of mitotic spindle microtubules to kinetochore	GO:0051315	4	1.86E-04
regulation of cytokinesis	GO:0032465	5	2.65E-04
metaphase plate congression	GO:0051310	4	2.65E-04
protein phosphorylation	GO:0006468	10	0.001
positive regulation of mitotic sister chromatid separation	GO:1901970	3	0.001
spindle assembly involved in female meiosis I	GO:0007057	3	0.001
DNA repair	GO:0006281	8	0.001
positive regulation of mitotic cytokinesis	GO:1903490	3	0.002
positive regulation of exit from mitosis	GO:0031536	3	0.003
CENP-A containing nucleosome assembly	GO:0034080	3	0.004
positive regulation of chromosome condensation	GO:1905821	3	0.004
mitotic cell cycle checkpoint	GO:0007093	3	0.004
positive regulation of attachment of mitotic spindle microtubules to kinetochore	GO:1902425	3	0.004
cell proliferation	GO:0008283	6	0.006
positive regulation of chromosome separation	GO:1905820	3	0.006
protein localization to kinetochore	GO:0034501	3	0.009
regulation of cyclin-dependent protein serine/threonine kinase activity	GO:0000079	4	0.009
**CELLULAR COMPONENTS (CCs)**			
kinetochore	GO:0000776	20	1.13E-22
chromosome, centromeric region	GO:0000775	13	1.30E-16
nucleoplasm	GO:0005654	47	1.39E-15
midbody	GO:0030496	16	1.40E-15
spindle	GO:0005819	15	1.50E-15
nucleus	GO:0005737	52	1.47E-12
microtubule cytoskeleton	GO:0015630	14	1.47E-12
cytosol	GO:0005829	50	1.47E-12
spindle microtubule	GO:0005876	9	2.49E-11
centrosome	GO:0005813	18	3.94E-11
condensed chromosome outer kinetochore	GO:0000940	6	2.57E-9
spindle pole	GO:0000922	10	4.97E-9
mitotic spindle	GO:0072686	10	8.29E-9

kinesin complex	GO:0005871	7	7,37E-03
mitotic spindle midzone	GO:1990023	5	6.82E-7
chromosome passenger complex	GO:0032133	4	3.73E-6
condensed chromosome	GO:0000793	5	1.08E-5
intercellular bridge	GO:0045171	6	6.17E-5
chromosome	GO:0005694	8	1.03E-4
Ndc80 complex	GO:0031262	3	2.41E-4
centriole	GO:0005814	6	7.82E-4
condensin complex	GO:0000796	3	9.96E-4
cyclin-dependent protein kinase holoenzyme complex	GO:0000307	4	0.001
nuclear chromosome	GO:0000228	4	0.003
chromocenter	GO:0010369	3	0.003
microtubule organizing center	GO:0005815	5	0.006
anaphase-promoting complex	GO:0005680	3	0.007
macromolecular complex	GO:0032991	9	0.007
**MOLECULAR FUNCTIONS (MFs)**			
microtubule binding	GO:0008017	16	3.15E-12
ATP-dependent microtubule motor activity	GO:1990939	9	1.14E-10
microtubule motor activity	GO:0003777	9	1.56E-9
ATP binding	GO:0005524	25	8.04E-9
chromatin binding	GO:0003682	11	1.24E-4
ATP-dependent microtubule motor activity, plus-end-directed	GO:0008574	4	4.84E-4
protein serine/threonine kinase activity	GO:0004674	9	0.001
protein kinase binding	GO:0019901	10	0.001
ATPase activity	GO:0016887	8	0.003
